# The periaqueductal gray in chronic low back pain: dysregulated neurotransmitters and function

**DOI:** 10.1097/j.pain.0000000000003617

**Published:** 2025-05-15

**Authors:** Laura Sirucek, Iara De Schoenmacker, Lindsay Mary Gorrell, Robin Lütolf, Anke Langenfeld, Mirjam Baechler, Brigitte Wirth, Michèle Hubli, Niklaus Zölch, Petra Schweinhardt

**Affiliations:** a Department of Chiropractic Medicine, Balgrist University Hospital, University of Zurich, Zurich, Switzerland; b Neuroscience Center Zurich, University of Zurich, Zurich, Switzerland; c Center for Neuroplasticity and Pain (CNAP), Department of Health Science and Technology, Aalborg University, Aalborg, Denmark; d Spinal Cord Injury Center, Balgrist University Hospital, University of Zurich, Zurich, Switzerland; e Biomedical Data Science Lab, Institute of Translational Medicine, Swiss Federal Institute of Technology (ETH) Zurich, Zurich, Switzerland; f Department of Forensic Medicine and Imaging, Institute of Forensic Medicine, University of Zurich, Zurich, Switzerland; g Department of Psychiatry, Psychotherapy and Psychosomatics, Psychiatric Hospital, University of Zurich, Zurich, Switzerland

**Keywords:** Brainstem, Chronic pain, Descending pain modulation, Excitatory/inhibitory balance, GABA, Glutamate, Magnetic resonance spectroscopy

## Abstract

Mechanisms underlying chronic pain are insufficiently understood, hampering effective treatment approaches. Preclinical evidence suggests a potential contribution of decreased excitatory (glutamatergic) and increased inhibitory (γ-aminobutyric acid [GABA]ergic) neurotransmission in the periaqueductal gray (PAG), a key descending pain modulatory brainstem area. This magnetic resonance spectroscopy (MRS) study investigated (1) whether a lower excitatory/inhibitory balance is also observed in the PAG of patients with nonspecific chronic low back pain (CLBP) and (2) whether the excitatory/inhibitory balance relates to psychophysical measures of descending pain modulation and pain sensitivity. Magnetic resonance spectroscopy was acquired on a 3T MR system in 41 patients with CLBP and 29 age- and sex-matched controls. Descending pain modulation and pain sensitivity were evaluated using conditioned pain modulation and pressure pain stimuli, respectively, which were both assessed at the lower back as the most painful area and the nondominant hand as a pain-free, remote area. Patients with CLBP presented with a lower glutamate + glutamine (Glx)/GABA ratio compared with controls (*P* = 0.002), driven by both decreased Glx (*P* = 0.012) and increased GABA (*P* = 0.038). Controls with lower Glx/GABA were more sensitive to pressure pain in both areas, but this association was missing in the patients (lower back: *P* = 0.004; hand: *P* = 0.002). Patients with more severe clinical pain showed impaired descending pain modulation at the hand (*P* = 0.003). In line with preclinical evidence, these findings support a dysregulated PAG in patients with CLBP that might be associated with dysfunctional descending pain inhibition.

## Abstract 2

Supplemental Digital Content is Available in the Text.

The excitatory/inhibitory balance in the periaqueductal gray of chronic low back pain patients was reduced and, contrary to pain-free participants, not associated with pain sensitivity.

## 1. Introduction

Chronic pain mechanisms are insufficiently understood, posing a challenge to the development of effective treatment options. Preclinical evidence suggests excitatory glutamatergic/inhibitory γ-aminobutyric acid (GABA)ergic imbalances in pain-relevant brain regions as a potential contributor to chronic pain.^37^ In patients with chronic pain, alterations of glutamate, glutamine, glutamate + glutamine (Glx), or GABA have been demonstrated in pain-relevant brain regions using proton magnetic resonance spectroscopy (^1^H-MRS).^60^

One brain region in which excitatory/inhibitory imbalances might have a substantial impact on pain processing is the periaqueductal gray (PAG). This key descending pain modulatory brainstem region^4^ exerts pain inhibition or facilitation through descending projections to the rostral ventromedial medulla and the spinal cord. Based on the “GABA disinhibition” hypothesis,^4^ PAG-driven descending pain inhibition is activated by turning off tonic GABAergic controls of descending glutamatergic projections. Conceivably, an excessive GABAergic tone in the PAG might impede its inhibitory function and contribute to aberrant pain processing. A reduced glutamatergic tone could have a similar effect because glutamate can indirectly inhibit GABAergic controls in the PAG^51^ and produce PAG-driven analgesia.^5^ In line with these notions, an augmented GABAergic tone^25^ as well as a hypo-glutamatergic state^29^ have been observed in the PAG of animal models of chronic neuropathic pain. In humans, investigations of PAG neurochemistry have been emerging more frequently in recent years, although they remain relatively scarce.^10,12,32,38,69,76^ This is possibly due to ^1^H-MRS in the PAG being technically challenging given the PAG's small size and high levels of physiological noise.^9^ Existing PAG ^1^H-MRS studies have addressed these challenges by using long echo times, which reduces the influence from macromolecules and lipids on the spectrum benefitting reliable metabolite detection, but hampers detection of J-coupled metabolites such as Glx or GABA,^38^ or by using large volumes of interest (VOI), which increases signal-to-noise ratios (SNR) but limits regional specificity.^10,12,32,76^

In this study, we adopted an improved approach enabling high-quality ^1^H-MRS acquisition in the PAG^71^ with the aim to investigate neurochemical alterations in patients with nonspecific chronic low back pain (CLBP). In brief, Glx and GABA were measured in a 1.1 mL small VOI using a Point-RESolved Spectroscopy sequence (PRESS)^7,58^ combined with Very Selective Saturation (VSS) pulses (OVERPRESS)^20,68,78^ and voxel-based flip angle calibration^59^ to optimize MRS acquisition, and with spectral registration to optimize MRS preprocessing.^53^ This sequence is not explicitly optimized for GABA quantification but allowed a high regional specificity. The PAG's excitatory/inhibitory balance, conceptualized as Glx/GABA, was compared between patients with CLBP and pain-free controls. Lower Glx/GABA, ie, decreased Glx and/or increased GABA, was expected in CLBP patients. In addition, it was investigated whether Glx/GABA was related to a psychophysical measure of descending pain modulation, ie, conditioned pain modulation (CPM).^80^ Lower Glx/GABA was expected to be associated with smaller CPM effects indicative of weaker descending pain inhibitory capacity. Finally, associations of Glx/GABA with experimental pressure pain sensitivity, and associations of Glx/GABA and CPM effects with clinical characteristics were explored.

## 2. Materials and methods

### 2.1. Participants

Chronic low back pain patients were consecutively recruited through the Balgrist University Hospital and advertisements in Swiss Chiropractic practices and patient magazines. Individually age- and sex-matched, pain-free controls were recruited through online advertisements and oral communication. Participants were recruited between October 2019 and February 2022. Data were collected between December 2019 and April 2022. The sample size calculation is described in Supplementary Methods M1, http://links.lww.com/PAIN/C268. Inclusion criteria for CLBP patients were between 18 and 80 years of age and CLBP as the primary pain complaint without signs or symptoms of serious underlying pathology (eg, infection, fractures, or spondyloarthropathies) or radiculopathy (ie, motor and sensory deficits) and of a duration longer than 3 months. For controls, the same inclusion criteria were applied with the additional requirement that they had not experienced low back pain lasting longer than 3 consecutive days during the past year. Considering the high prevalence of low back pain,^49^ it is possible that they have experienced low back pain at an earlier point in their life or low back pain with a shorter duration than 3 consecutive days during the past year. Exclusion criteria for both cohorts comprised any major medical or psychiatric condition other than CLBP, pregnancy, inability to follow study instructions, or contraindications to MRI. The study was approved by the local ethics committee “Kantonale Ethikkommission Zürich” (Nr.: 2019-00136), registered on clinicaltrials.gov (NCT04433299), and performed according to the guidelines of the Declaration of Helsinki (2013). Written informed consent was obtained from all participants before the start of the experiment.

### 2.2. Study design

**Figure 1.**
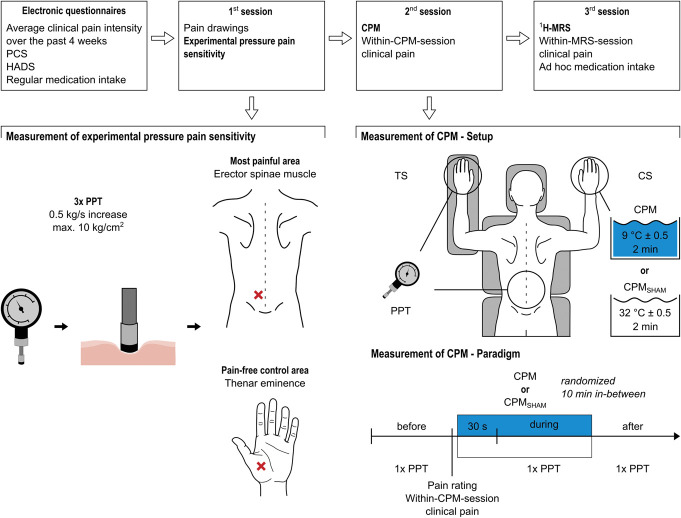
Overview of the study design and the CPM and experimental pressure pain sensitivity assessment. Main outcomes of interest are highlighted in bold. Red crosses indicate locations at which PPTs were assessed, ie, over the erector spinae muscle within the lower back (most painful area) and at the thenar eminence of the nondominant hand (pain-free, remote area). The CPM_SHAM_ paradigm was only performed at the nondominant hand. CPM, conditioned pain modulation; CS, conditioning stimulus; ^1^H-MRS, proton magnetic resonance spectroscopy; HADS, hospital anxiety and depression scale; PCS, pain catastrophizing scale; PPT, pressure pain threshold; TS, test stimulus.

This study was part of a larger project (Clinical Research Priority Program “Pain,” https://www.crpp-pain.uzh.ch/en.html), which comprised 3 experimental sessions of approximately 3 hours each and electronic questionnaires. In the first 2 sessions, participants underwent clinical, neurophysiological, and psychophysical assessments. The CPM assessment was always performed in the second session preceded by the acquisition of pain-evoked potentials. During the third session, participants underwent 2 MR measurements, 1 ^1^H-MRS scan and 1 resting-state functional MRI scan with a break of 1 hour between. All scans were performed after 12 pm. This study concerns the ^1^H-MRS, the CPM, the pain drawings, the experimental pressure pain sensitivity of the first session's psychophysical assessment, and questionnaire data. The functional MRI data will be presented in a separate article. Figure 1 shows an overview of the study design.

### 2.3. Magnetic resonance spectroscopy

Substantial material in this section is identical to a technical note focused on methodological aspects of high-quality ^1^H-MRS acquisition in the PAG using the data of the controls.^71^ Technical details of the ^1^H-MRS are listed in Supplementary Table 1, http://links.lww.com/PAIN/C268. The following sections provide an overview of the applied methods.

#### 2.3.1. Acquisition

**Figure 2.**
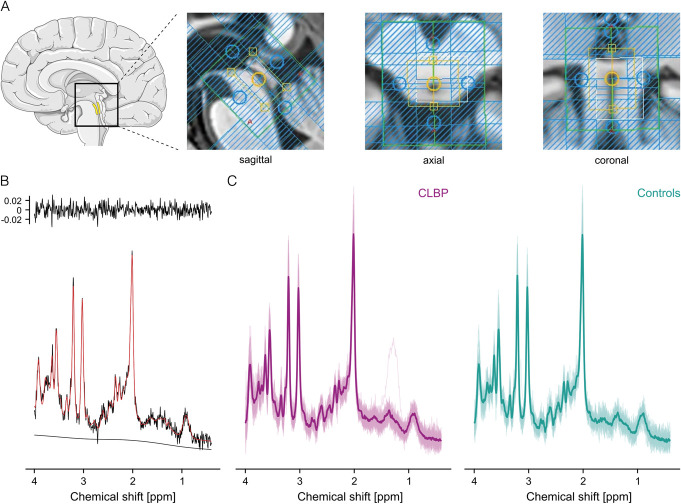
^1^H-MRS VOI placement and acquired spectra. (A) The VOI was placed according to anatomical landmarks such as the cerebral aqueduct. The yellow box represents the nominal size of the VOI (11 × 15 × 18 mm^3^ [AP × LR × FH]). Blue-shaded areas represent the VSS bands. The unshaded area within the yellow box represents the final VOI size (8.8 × 10.2 × 12.2 mm^3^). (B) Representative single spectrum (SNR = 19, FWHM H_2_O = 5.1 Hz, FWHM NAA = 4.9 Hz, age = within 53.5 years [median age of the cohort] ± 5). (C) Overlaid single spectra together with the group average (bold) for CLBP patients (purple) and controls (turquoise). The schematic brain was adapted from Servier Medical Art (smart.servier.com). AP, anterior–posterior; CLBP, nonspecific chronic low back pain; FWHM, full width at half maximum; FH, foot–head; ^1^H-MRS, proton magnetic resonance spectroscopy; LR, left–-right; NAA, N-acetylaspartate; SNR, signal-to-noise ratio; VOI, volume of interest; VSS, very selective saturation.

^1^H-MRS was acquired on a 3T MR system using a 32-channel head coil (Philips Healthcare, Best, The Netherlands). Before ^1^H-MRS acquisition, high-resolution (1 mm^3^ isotropic) anatomical T_1_-weighted images were obtained (acquisition time: 7 minutes 32 seconds). Based on the 3D T_1_ images, the VOI was placed to cover the PAG according to anatomical landmarks by the same examiner (LS) for all participants (Fig. 2A). Spectra were localized using a water-suppressed single-voxel OVERPRESS sequence^7,20,58,68,78^ (repetition time [TR]: 2500 ms; echo time [TE]: 33 ms; number of signals averaged: 512 divided into 8 blocks of 64; acquisition time: 23 minutes 20 seconds). The use of VSS pulses minimizes errors in chemical-shift displacement and allows for consistent localization volumes across all metabolites of interest (Fig. 2A). The voxel-based flip angle calibration^19,59^ achieves an optimal flip angle within the VOI. Accounting for the VSS pulses, the resulting VOI size was 8.8 × 10.2 × 12.2 mm^3^ = 1.1 mL. Although the used sequence is not explicitly optimized for GABA quantification, it allowed achieving a sufficient SNR in a VOI similar in size to the PAG within a reasonable scanning time, while being less susceptible to motion artifacts compared to sequences optimized for GABA quantification, i.e., MEscher-GArwood-PRESS (MEGA-PRESS).^48^ For each individual, 2 water signals were acquired: 1 obtained from interleaved water unsuppressed spectra (1 before each of the 8 blocks) for eddy current correction and internal water referencing corrected for partial volume and tissue-specific relaxation effects using literature-based T_1_ and T_2_ relaxation times (literature-based approach)^54^; 1 measured in a separate water reference scan after the ^1^H-MRS acquisition in the PAG within the same VOI with a TR of 10,000 milliseconds and varying TEs (33/66/107/165/261/600 ms), allowing estimation of the T_2_ relaxation time of water within the VOI. Hereby, a literature-independent, subject-specific approximation of the fully relaxed water signal was obtained (subject-specific approach).^36^

For a subset of participants, head motion was measured using the markerless motion tracking system Tracoline TCL3 with the TracSuite software 3.1.9 (TracInnovations, Ballerup, Denmark,) used for retrospective motion correction of positron emission tomography scans^56,72^ and prospective real-time motion correction of MRI scans.^6,72^ A more detailed description of the Tracoline system is provided in Supplementary Figure 1, http://links.lww.com/PAIN/C268 and in the previously published technical note on high-quality ^1^H-MRS acquisition in the PAG using the data of the controls.^71^ From the head motion recording, the head motion variability during the ^1^H-MRS scan and the head displacement between the anatomical T_1_ acquisition and the ^1^H-MRS acquisition were calculated and compared between cohorts using Wilcoxon rank-sum tests.

#### 2.3.2. Preprocessing and analysis

The T_1_-weighted images were segmented into gray matter (GM), white matter (WM), and cerebrospinal fluid (CSF) to obtain the respective tissue fractions (in %) within the VOI using SPM12.

Frequency alignment was performed using spectral registration in the time domain (adopted from Refs. 53,^70^). For that, the data were filtered with a 2-Hz Gaussian filter. Only the first 500 milliseconds were used for alignment, and the single averages were aligned to the median of all averages. Preprocessing was performed by an author blinded to the hypotheses of the study (N.Z.).

The preprocessed spectra were inspected for artefacts. Spectra with artefacts were excluded from further analyses, as were spectra presenting with insufficient quality,^59^ ie, with a full width at half maximum (FWHM) value of the unsuppressed water peak (FWHM H_2_O; shim quality indicator) above 2.5 median absolute deviation (MAD)^39^ of the group median or with a SNR below 2.5 MAD of the group median.

#### 2.3.3. Metabolite quantification

Spectra were analyzed using LCModel (6.3) with a metabolite basis set simulated using FID-A taking into account spatial localization and actual radiofrequency pulse shapes.^70^ Metabolite concentrations are reported as ratios to the unsuppressed water signal and reflect an estimation of metabolite concentration in moles per kg of tissue water excluding water within CSF. As primary outcome, the subject-specific water signal was used. In addition to being independent of literature-based tissue-specific T_1_ and T_2_ relaxation times, known to vary across brain regions^79^ and possibly not generalizable to the PAG, the subject-specific approach relies less on the correct segmentation of GM, WM, and CSF compared with the literature-based approach. For comparison, all analyses were also performed using the literature-based water signal.

### 2.4. Conditioned pain modulation

Conditioned pain modulation is a psychophysical measure of descending pain modulation reflecting how one noxious stimulus (test stimulus [TS]) is modulated (typically inhibited) by another, heterotopic noxious stimulus (conditioning stimulus [CS]).^80^ Participants were tested in a prone position in a quiet room (temperature 20-25°C). The CPM assessment was performed by a trained experimenter blinded to the hypotheses of the study. Participants were given standardized verbal instructions.

Conditioned pain modulation was assessed at the patient's most painful area in the lower back and at the nondominant hand as a pain-free, remote area. The order of the 2 areas was randomized with a balanced order between the cohorts (most painful area assessed first: 58.5% of CLBP patients, 51.7% of controls). The exact area on the lower back, the laterality of the hand (right or left), and the order of the 2 areas in each control were identical to the patient to whom they were individually age- and sex-matched.

The CS consisted of a circulating cold water bath (9°C ± 0.5) in which the participants immersed their dominant hand up to the wrist for 2 minutes. The perceived pain intensity of the CS was rated on a numeric rating scale (NRS) from 0 “no pain” to 10 “maximum pain tolerable” directly after immersion, 30 seconds after immersion and immediately before withdrawal of the hand. If the pain became intolerable, participants were allowed to withdraw their hand but were encouraged to re-immerse their hand as soon as possible.

The TS consisted of pressure pain thresholds (PPTs) assessed before, during (30 seconds after CS onset; “parallel CPM effect”), and after (“sequential CPM effect”) the CS. Pressure pain thresholds were chosen as TS because (1) deep afferents are likely to play a more important role compared with superficial afferents in CLBP as a musculoskeletal pain condition, and (2) the combination with a cold water bath as CS has been shown to be the CPM paradigm with the highest intrasession reliability^55^ and with larger CPM effects compared with other TS-CS combinations.^30^ Of note, CPM is based on diffuse noxious inhibitory controls (DNIC) observed in animals, and the first studies translating DNIC to humans used suprathreshold TS.^8^ However, DNIC have been shown to inhibit not only suprathreshold stimulation but also light touch^2^ and cuff pressure stimulation with intensities ramping up from 0 kPa to 40 kPa.^18^ Therefore, PPTs are considered a suitable TS for CPM paradigms. Pressure was applied using a hand-held mechanical algometer (Wagner Instruments, Greenwich, CT) with a circular rubber tip (1 cm diameter). For the lower back, PPTs were assessed over the erector spinae muscle at the level and the body side of the most painful area. For the hand, PPTs were assessed at the thenar eminence. One PPT per timepoint was determined by increasing the pressure at a rate of 0.5 kg/s until participants reported a change in perception from pressure alone to pain.^65^ If no pain was reported at 10 kg/cm^2^ (safety cut-off to avoid tissue damage), a value of 11 kg/cm^2^ was assigned as the PPT. Three additional test stimuli were administered, ie, pressure temporal summation of pain (TSP), heat pain thresholds, and heat TSP. The order of pressure and heat assessments was randomized with a balanced order between the cohorts (pressure assessed first: 53.7% of CLBP patients, 55.2% of controls), and TSP was always performed after the threshold assessments. These test stimuli answer a different research question and will not be discussed further; relevant here is that the paradigm was identical between the CLBP patients and the controls.

At the hand, an additional CPM_SHAM_ paradigm was performed to test whether the used CPM paradigm induced a “true” CPM effect beyond effects unrelated to the painfulness of the CS, eg, repeated-measures effects.^33^ The CPM_SHAM_ paradigm was performed identically to the CPM paradigm except for an ambient temperature water bath (32°C ± 0.5) as CS. Within the assessment of the hand, the order of CPM and CPM_SHAM_ paradigms was randomized with a balanced order between the cohorts (CPM paradigm assessed first: 51.2% of CLBP patients, 48.3% of controls) with a minimum of 10 minutes between.

### 2.5. Clinical characteristics

As part of the electronic questionnaires, CLBP patients reported their *average clinical pain intensity* over the past 4 weeks (NRS: 0 “no pain” to 10 “maximum pain”). To allow for differentiation between this “trait” pain and the “state” pain during the experimental sessions, 2 additional pain intensity ratings were acquired: (1) *within-MRS-session clinical pain*, where after the ^1^H-MRS scan, CLBP patients rated the maximum pain they had experienced during the scan (NRS: 0 “no pain” to 10 “most intense pain tolerable”) and (2) *within-CPM-session clinical pain,* where CLBP patients reported the current pain intensity in their most painful area before the first water bath on the same NRS as for (1).

Chronic low back pain patients completed pain drawings before the first experimental session to assess the spatial pain extent (% of the total body area) of their typically painful body areas.^66,67^ Participants were instructed to shade their typically painful areas on printed standardized body charts (frontal and dorsal view). After manual contouring of the shaded areas, the pain drawings were digitized and processed using a custom-made software that calculated the spatial extent of the shaded areas as a percentage of the total body area. Only LBP-associated areas, ie, the lower back, the buttocks, and the legs, were considered for further analyses.

Participants also completed the Pain Catastrophizing Scale (PCS)^73^ and the Hospital Anxiety and Depression Scale (HADS).^85^ The PCS consists of 13 items with a score range between 0 and 52 (>30 clinically relevant level of catastrophizing). The HADS consists of 14 items with a 7-item anxiety subscale and a 7-item depression subscale, with a score range between 0 and 21 per subscale (8-10 moderate and 11-21 high probability for a mood disorder).

### 2.6. Confounding factors

Before the CPM and the ^1^H-MRS session, information about the menstrual cycle phase and medication intake was obtained (Supplementary Methods M2, http://links.lww.com/PAIN/C268).

### 2.7. Statistical analysis

All statistical analyses were performed in RStudio for Mac (2022.12.0 + 353) and for Windows (2024.09.0 + 375). Statistical significance was set at *α* = 0.05 with a false discovery rate (FDR) correction per tested research question. The number of corrected tests per research question is indicated as *n*-FDR.

Depending on the statistical test used, raw values or model residuals were assessed for normal distribution using inspection of histograms and QQplots. All ordinal or non-normally distributed variables are reported as median (interquartile range) and were analyzed using nonparametric tests. All continuous variables that met normality assumptions are reported as mean (SD) and were analyzed using parametric tests.

Group comparisons were performed using linear models (Supplementary Methods M3, http://links.lww.com/PAIN/C268). This allowed for the examination of the potential influences of age and sex on the dependent variables and of influential cases in a standardized manner (Supplementary Methods M4, http://links.lww.com/PAIN/C268). For all models, the number of identified influential cases is indicated as *n*-IC. If removal of influential cases changed the statistical inferences, the results of the model without the influential cases are reported (indicated as *n*-IC^†^). Otherwise, the results of the full data set are reported. Assessing influential cases ensured that the obtained results were not driven by a small number of extreme datapoints. If linear model assumptions were not met, a suitable alternative was used (Supplementary Methods M3, http://links.lww.com/PAIN/C268).

Effect sizes are reported using *partial η*^*2*^ (small: 0.01; medium: 0.06; large: 0.14) for linear models, Cohen *d* (small: <0.5; medium: 0.5-0.8; large: >0.8) for *t*-tests, and *r* (small: 0.1-<0.3; medium: 0.3-<0.5; large: ≥0.5) for Wilcoxon rank-sum tests. No effect sizes are reported for linear mixed models because no agreement on standard effect sizes exists.^63^

#### 2.7.1. Magnetic resonance spectroscopy

Group comparisons were performed for: (1) Glx/GABA as the main outcome of interest; (2) Glx and GABA separately to examine which metabolite drove the observed group difference in Glx/GABA (*n*-FDR: 2); and (3) tCr (creatine + phosphocreatine), tCho (glycerophosphocholine + phosphocholine), tmI (myo-inositol + glycine), tNAA (N-acetylaspartate + N-acetylaspartylglutamate), as well as GM, WM, and CSF tissue fractions. For (3), no multiple comparison correction was performed because it was not part of the primary research question.

Because differences in Glx or GABA might be confounded by differences in GM or WM tissue fractions, it was investigated whether Glx or GABA correlated with GM or WM tissue fractions in the controls, ie, a “healthy” state, using Pearson correlations (without multiple comparison correction to minimize the risk for false negatives). An alternative approach to account for GM and WM tissue fractions for GABA quantification is to correct GABA estimates for an assumed ratio (α) between the GABA concentrations in WM and GM.^27^ However, for this study, this approach was not deemed suitable because (1) assumptions underlying the α correction factor are strong, ie, it is not established which α value represents the ground truth, especially for the scarcely investigated PAG; (2) α correction has so far been suggested only for GABA^61^ but not for Glx or other metabolites,^54^ making its application to metabolites other than GABA in this study unsubstantiated; (3) it is conceivable that pathologies may influence the neurotransmitter distribution across GM and WM, and applying the same α correction factor to both controls and patient cohorts might introduce an unintended confounder to group comparisons.

Furthermore, GABA quantification could be influenced by how the baseline, macromolecules, or lipids were fitted in the spectra. To exclude such influences on this study results related to GABA, spectral baseline and macromolecule/lipid fit were compared between the cohorts (Supplementary Methods M5, http://links.lww.com/PAIN/C268).

#### 2.7.2. Conditioned pain modulation

Conditioned pain modulation effects were calculated as follows: parallel CPM effect = PPT before − PPT during, sequential CPM effect = PPT before − PPT after. Thus, inhibitory CPM effects are denoted by a negative value and facilitatory CPM effects by a positive value. For all *within-subject* analyses, absolute values were used. For all *between-subject* and correlational analyses, relative differences, ie, ((PPT before − PPT during)/PPT before) × 100 were used to allow direct comparisons between the lower back and the hand (which might have different PPTs). For CPM inhibition, values exceeding −100% were set to −100%, so that the magnitude of maximum possible CPM inhibition was identical to the magnitude of maximum possible CPM facilitation (ie, +100%).

##### 2.7.2.1. Within-subject analyses

First, the presence of “true” CPM effects beyond effects unrelated to the painfulness of the CS was tested on the data of the controls using 2 Wilcoxon signed-rank tests (*n*-FDR: 2) because assumptions for a linear mixed model were not met. All subsequent analyses were performed using the detected “true” CPM effect, ie, the CPM timepoint which showed a difference between the CPM and the CPM_SHAM_ paradigm.

Second, it was analyzed whether this “true” CPM effect was present in both cohorts in both areas. For that, 4 linear mixed models were performed (Supplementary Methods M3, http://links.lww.com/PAIN/C268) (*n*-FDR: 2 per cohort).

##### 2.7.2.2. Between-subject analyses

Group comparisons were performed for “true” CPM effects in both areas (*n*-FDR: 2).

In addition, participants were classified into CPM inhibitors (PPT increase during CPM > 2 standard error of measurement [SEM] of PPTs), CPM facilitators (PPT decrease > 2 SEM) or CPM nonresponders (PPT increase/decrease ≤ 2 SEM)^33^ (Supplementary Methods M6, http://links.lww.com/PAIN/C268). Proportions of CPM inhibitors, CPM facilitators and CPM nonresponders in both areas were compared between the cohorts using Fisher exact tests (*n*-FDR: 2).

#### 2.7.3. Associations of Glx/GABA with conditioned pain modulation effects and experimental pressure pain sensitivity

Associations of Glx/GABA with “true” CPM effects and experimental pressure pain sensitivity were tested using linear models because this allowed to test for “cohort” interaction effects, ie, whether the associations differed between the cohorts. Associations were examined for both areas (*n*-FDR: 2 for “true” CPM effects and 2 for experimental pressure pain sensitivity). The analyses on experimental pressure pain sensitivity were also performed for Glx and GABA separately in an exploratory manner (ie, without multiple comparison correction).

Pressure pain thresholds were used as a proxy for experimental pressure pain sensitivity. Here, PPTs from the first experimental session of the larger project were used to avoid a flawed analysis because a random string B-A (here the “true” CPM effect) will typically correlate with a random string A (here PPTs before CPM). In the first experimental session, for each testing area, 3 PPTs were acquired.^65^ The average PPT of each area was used for analysis.

In addition, 2 Spearman correlations were performed to explore whether the “true” CPM effects depended on experimental pressure pain sensitivity in the controls, ie, a “healthy” state.

#### 2.7.4. Associations of Glx/GABA and conditioned pain modulation effects with clinical characteristics

In CLBP patients, Spearman correlations were used to investigate associations of Glx/GABA and “true” CPM effects in both areas with clinical characteristics, ie, (1) average clinical pain intensity over the past 4 weeks, (2) pain duration (in months), (3) spatial pain extent, and (4) within-MRS-session clinical pain or within-CPM-session clinical pain (*n*-FDR: 4 for associations with Glx/GABA; *n*-FDR: 8 for associations with “true” CPM effects). For clinical characteristics that correlated with CPM effects, additional Spearman correlations with PPTs from the first experimental session were performed to examine whether observed association of clinical characteristics with CPM effects did not, in fact, represent associations of clinical characteristics with experimental pressure pain sensitivity. Using 3 additional Spearman correlations, associations of Glx/GABA with indicators of psychological distress in the CLBP patients, ie, PCS and HADS anxiety and depression scores, were explored without multiple comparison correction because of the exploratory nature of this analysis. The same analyses were performed for Glx and GABA separately in an exploratory manner (ie, without multiple comparison correction).

#### 2.7.5. Confounding factors

The statistical analyses of influences of menstrual cycle phases and medication intake on Glx/GABA or the parallel CPM effects are described in the Supplementary Methods M2, http://links.lww.com/PAIN/C268.

## 3. Results

For all linear models and Welch *t* tests, full model results, including information on age, sex, and influential cases effects, are presented in Supplementary Table 2, http://links.lww.com/PAIN/C268. If not mentioned otherwise, there were no effects of age or sex on the analyzed dependent variables.

### 3.1. Participants

Of 83 recruited participants (48 CLBP patients and 35 controls), 3 (CLBP patients) cancelled before the first session, 3 (2 CLBP patients and 1 control) were excluded due to a suspected neurological or psychiatric disorder, and 1 (control) discontinued the scanning session because of discomfort. Five additional participants (2 CLBP patients and 3 controls) were excluded due to artefacts in the MR spectra (Supplementary Figure 2, http://links.lww.com/PAIN/C268). One additional control was excluded due to insufficient MRS quality (low SNR, see section Magnetic resonance spectroscopy, Preprocessing and analysis).

The demographics of the final sample (41 CLBP patients and 29 controls) are described in Table 1.

**Table 1:** Participant demographics and clinical characteristics.

	CLBP patients (n = 41)	Controls (n = 29)	Test statistic	*P*	Effect size
Age (y)	54 (41-65)	47 (34-67)	*W* = 553	0.625	*r* = 0.06
Sex (female:male) [n]	22:19	17:12		0.810*	
BMI (kg/m^2^)	24.5 (3.47)	23.3 (2.60)	*t* = 1.5	0.128	*d* = −0.37
Educational level [n (%)]					
Primary education	2 (4.9)	0 (0)		0.236*	
Upper secondary education	3 (7.3)	3 (10.3)			
Short-cycle tertiary education	24 (58.5)	11 (26.8)			
At least Bachelor's or equivalent level	12 (29.3)	15 (36.6)			
PCS	**11 (5-** **22)**	**3 (0-** **8)**	***W*** ** = 267.5**	**<0.001**	***r*** ** = 0.49**
HADS anxiety	4 (2-7)	3 (1-5)	*W* = 485.5	0.193	*r* = 0.16
HADS depression	**3 (1-** **6)**	**1 (0-** **2)**	***W*** ** = 314**	**<0.001**	***r*** ** = 0.41**
Average clinical pain intensity (NRS)	4 (3.0-5.0)				
Pain duration [mo]	106 (17.0-205.3)†				
Spatial pain extent (%)	1.3 (0.50-2.20)				
Within-MRS-session clinical pain (NRS)	1.5 (0-4.0)				
Within-CPM-session clinical pain (NRS)	2 (0-4.0)				

Values are presented as mean (SD) for continuous variables and as median (interquartile range) for ordinal or non-normally distributed variables. *W*-statistics refer to Wilcoxon rank-sum tests and *t*-statistics to unpaired *t-*tests. Bold values indicate the statistically significant results.

* Fisher exact test.

† N = 40 due to 1 missing value (participant did not indicate the month of pain onset).

BMI, body mass index; CLBP, nonspecific chronic low back pain; CPM, conditioned pain modulation; HADS, hospital anxiety and depression scale; MRS, magnetic resonance spectroscopy; NRS, numeric rating scale; PCS, pain catastrophizing scale.

### 3.2. Magnetic resonance spectroscopy

The acquired ^1^H-MR spectra were of high quality as indicated by narrow linewidths of the acquired unsuppressed water peak (FWHM H_2_O) and adequate SNR (Table 2, Figs. 2B and C). Importantly, quality measures did not differ between the cohorts (Table 2). All metabolites were detected with adequate accuracy, ie, mean Cramér-Rao lower-bounds (CRLB) below 20% (Supplementary Table 3, http://links.lww.com/PAIN/C268). For GABA, individual absolute^35^ and relative CRLBs are reported in Supplementary Table 4, http://links.lww.com/PAIN/C268. Head motion was tracked in 30 CLBP patients and 25 controls. Neither head motion variability (median [IQR] for patients: 0.6 mm [0.41-0.85]; controls: 0.5 mm [0.33-0.74]) nor head displacement (patients: 1.1 mm [0.50-1.96]; controls: 1.7 mm [0.84-1.98]) was different between cohorts (*W* = 333, *P* = 0.647, *r* = 0.06 and *W* = 415, *P* = 0.346, *r* = 0.13).

**Table 2:** Proton magnetic resonance spectroscopy outcomes, experimental pressure pain sensitivity, and conditioned pain modulation effects.

^**1**^ **H-** **MRS outcomes**	CLBP patients (n = 41)	Controls (n = 29)	*n*-IC	Test statistic	*P*	Effect size
SNR	19 (18-19)	18 (17-21)		*W* = 567.5	0.748	*r* = 0.04
FWHM H_2_O (Hz)	5.4 (0.91)	5.4 (0.70)		*t* = 0.3	0.737	*d* = 0.08
FWHM NAA (Hz)	4.3 (3.96-4.85)	4.9 (3.96-4.85)		*W* = 545	0.561	*r* = 0.07
Glx/GABA	**4.0 (1.05)**	**4.9 (1.17)**	1	***F*** ** = 10.7**	**0.002**	***η*** ^***2***^ ** = 0.14**
Glx (mmol/kg)	**8.9 (1.34)**	**9.9 (1.57)**	4	***F*** ** = 8.0**	**0.012** *	***η*** ^***2***^ ** = 0.11**
GABA (mmol/kg)	**2.4 (0.67)**	**2.1 (0.45)**	1^†^	***F*** ** = 4.5**	**0.038** *	***d*** ** = 0.46**
tCr (mmol/kg)	6.5 (0.57)	6.6 (0.46)	4	Not meaningful‡		
tCho (mmol/kg)	2.2 (0.24)	2.2 (0.18)	3	Not meaningful‡		
tmI (mmol/kg)	8.9 (0.91)	9.0 (1.03)		*F* = 0.2	0.661	*η*^*2*^ = 0.00
tNAA (mmol/kg)	9.1 (1.16)	9.0 (0.87)		*F* = 0.2	0.654	*η*^*2*^ = 0.00
GM (% of VOI)	52.7 (5.19)	50.2 (5.04)	2^†^	*F* = 4.0	0.050	*η*^*2*^ = 0.06
WM (% of VOI)	**41.2 (7.20)**	**45.0 (6.0)**	2	***F*** ** = 5.4**	**0.024**	***η*** ^***2***^ ** = 0.07**
CSF (% of VOI)	6.0 (3.9)	4.8 (2.5)	3	*W* = 510	0.312	*r* = 0.12

**Experimental pressure pain sensitivity**						
PPT LB (kg/cm^2^)	5.7 (2.76)	6.1 (2.51)		*t* = −0.61	0.539	*d* = 0.15
PPT hand (kg/cm^2^)	4.0 (2.97-5.20)	3.8 (2.83-4.90)		*W* = 553	0.625	*r* = 0.06

CPM effects	CPM_SHAM_ Controls (n = 29)	CPM Controls (n = 29)				
PPT before hand (kg/cm^2^)	4.4 (1.75)	4.2 (1.59)				
PPT during hand (kg/cm^2^)	4.5 (1.49)	5.1 (1.84)				
PPT after hand (kg/cm^2^)	4.6 (1.63)	4.6 (1.49)				
ΔPPT parallel hand (kg/cm^2^)	**0 (−** **0.60 to 0.60)**	**−** **1.1 (−** **1.50 to −** **0.50)**		***V*** ** = 278**	**0.004**	
ΔPPT sequential hand (kg/cm^2^)	−0.1 (−0.60 to 0.40)	−0.5 (−1.03 to 0.10)		*V* = 212.5	0.183	
ΔPPT parallel hand (%)	−5.4 (25.15)	−27.6 (33.40)				
ΔPPT sequential hand (%)	−7.1 (26.99)	−14.7 (26.39)				

	CLBP patients (n = 41)	Controls (n = 29)				
Cold water bath LB pain intensity (NRS)	9 (7-10)	8 (7-9)		*W* = 530	0.438	*r* = 0.09
Cold water bath hand pain intensity (NRS)	8 (6-9)	8 (7-9)		*W* = 572	0.791	*r* = 0.03
PPT before LB (kg/cm^2^)	5.6 (2.77)	6.3 (2.38)				
PPT during LB (kg/cm^2^)	6.9 (2.96)	8.0 (2.27)				
CPM ΔPPT parallel LB (kg/cm^2^)	**−1.2 (1.37)** §	**−1.6 (1.55)** §				
CPM ΔPPT parallel LB (%)	−25.8 (33.25)	−33.0 (34.15)	4	*F* = 0.75	0.388*	*η*^*2*^ = 0.01
PPT before hand (kg/cm^2^)	4.5 (2.09)	4.2 (1.59)				
PPT during hand (kg/cm^2^)	5.0 (2.0)	5.1 (1.84)				
CPM ΔPPT parallel hand (kg/cm^2^)	**−0.5 (0.95)** ‖	**−0.9 (1.21)** §				
CPM ΔPPT parallel hand (%)	−16.9 (24.83)	−27.6 (33.40)	3	*F* = 2.3	0.274*	*η*^*2*^ = 0.03

Values are presented as mean (SD) for continuous variables and as median (interquartile range) for ordinal or non-normally distributed variables, or data that did not meet requirements for parametric testing. *n*-IC indicates the number of identified influential cases in the respective model. *n*-IC^†^ indicates that removal of influential cases changed the statistical inference of the model. *W*-statistics refer to Wilcoxon rank-sum tests, *t*-statistics to unpaired *t* tests or Sidak-corrected post hoc tests (*t*^*ph*^), and *F*-statistics to linear models or Welch test for GABA. *η*^*2*^ values refer to *partial η*^*2*^'s. The number of missing values for each variable is reported in Supplementary Table 6, http://links.lww.com/PAIN/C268. Bold values indicate the statistically significant results.

* FDR corrected for n = 2 tests.

‡ Due to the presence of a significant “cohort X gender” interaction effect.

§ Statistically significant within-group CPM effect with *P* < 0.001, FDR corrected for n = 2 tests.

‖ Statistically significant within-group CPM effect with *P* < 0.01, FDR corrected for n = 2 tests.

CLBP, nonspecific chronic low back pain; CPM, conditioned pain modulation; CSF, cerebrospinal fluid; FDR, false discovery rate; FWHM, full width at half maximum; GABA, γ-aminobutyric acid; Glx, glutamate + glutamine; GM, gray matter; ^1^H-MRS, proton magnetic resonance spectroscopy;  LB, lower back; NRS, numeric rating scale; PPT, pressure pain threshold; SNR, signal-to-noise ratio; tCho, glycerophosphocholine + phosphocholine; tCre, creatine + phosphocreatine; tmI, myo-inositol + glycine; tNAA, N-acetylaspartate + N-acetylaspartylglutamate; VOI, volume of interest; WM, white matter.

**Figure 3.**
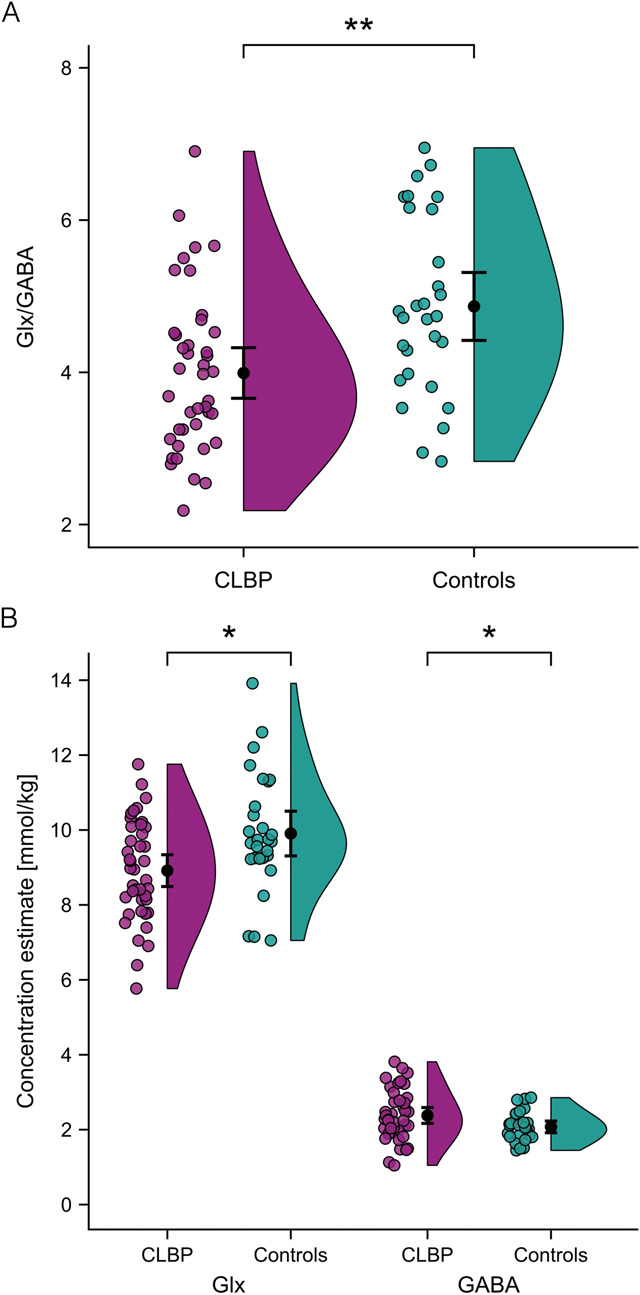
Lower Glx/GABA in CLBP patients driven by decreased Glx and increased GABA. The raincloud plots show the raw data (coloured dots), means and 95% confidence intervals (black dots and bars), and probability distributions (vertical “clouds”) of Glx/GABA (A) and Glx and GABA separately (B) for CLBP patients (purple) and controls (turquoise). *P < 0.05, **P < 0.01. CLBP, nonspecific chronic low back pain; GABA, γ-aminobutyric acid; Glx, glutamate + glutamine.

Glx/GABA was significantly lower in CLBP patients compared with controls (Table 2, Fig. 3A). This effect was driven by lower Glx as well as higher GABA in CLBP patients compared with controls (Table 2, Fig. 3B).

The cohorts did not differ in the remaining metabolites (Table 2) except for a sex effect on tCr and tCho in CLBP patients (Supplementary Table 2, http://links.lww.com/PAIN/C268) with highest concentrations observed in CLBP men. Across cohorts, tCr increased with age and tmI was higher in men (Supplementary Table 2, http://links.lww.com/PAIN/C268). There were no differences in GM or CSF tissue fractions between the cohorts (Table 2). However, CSF tissue fractions increased with age in CLBP patients (*rho* = 0.73, *P* < 0.001) but not in controls (*rho* = 0.04, *P* = 0.831). Furthermore, patients presented with lower WM tissue fractions compared with controls (Table 2).

Observed group differences in Glx or GABA were independent of variations in GM or WM tissue fractions because within controls, neither Glx nor GABA correlated with GM (Glx: *r* = −0.23, *P* = 0.224; GABA: *r* = 0.04, *P* = 0.822) or WM tissue fractions (Glx: *r* = 0.32, *P* = 0.091, GABA: *r* = 0.10, *P* = 0.617).

The observed GABA differences between cohorts were unlikely due to differences in spectral baseline or macromolecule/lipid fit, both of which can potentially influence GABA quantification (Supplementary Results R1, Supplementary Figure 3, http://links.lww.com/PAIN/C268).

Qualitatively similar cohort effects were observed using the literature-based water signals (Supplementary Table 5, http://links.lww.com/PAIN/C268).

### 3.3. Conditioned pain modulation

Cold water baths induced moderate to intense pain (Table 2). Six participants (5 CLBP and 1 control) reported low pain (NRS 1-3) in 1 or 2 cold water baths. Seven participants (5 CLBP and 2 controls) did not tolerate the full 2 minutes of 1 or 2 cold water baths. Two controls reported pain (NRS 1 and 2) during the ambient temperature water bath. For 1 CLBP patient and the respective matched control, CPM and experimental pressure pain sensitivity were assessed at the upper arm as the pain-free, remote area because of scarring at the CLBP patient's hand. These cases did not have an influence on the results because they were not identified as influential cases that changed the statistical inference of a model. Sequential CPM effects were assessed on average 2 minutes 58 seconds (SD = 49 seconds) after CS end.

**Figure 4.**
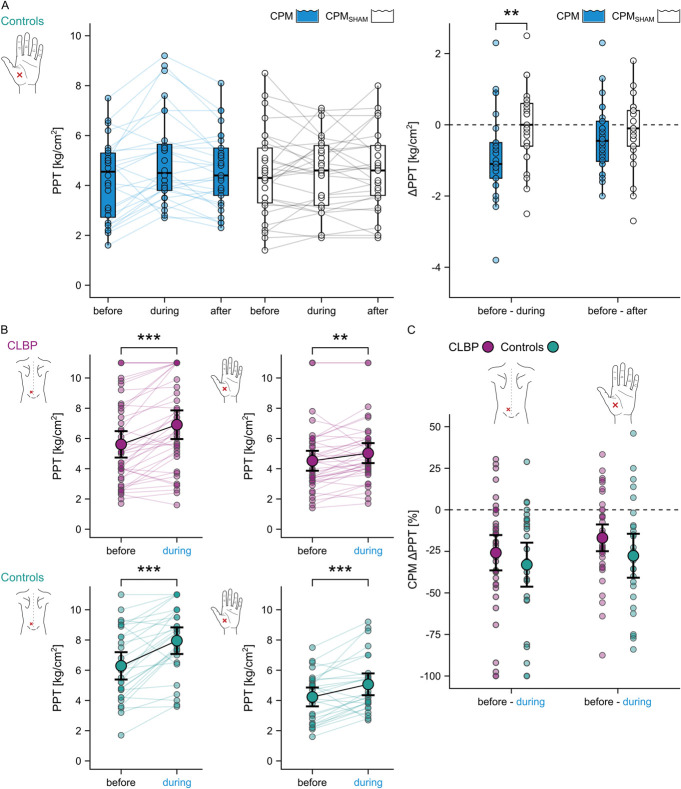
Inhibitory CPM effects on PPTs in CLBP patients and pain-free controls. (A) PPT changes in controls in response to the CPM (blue) and the CPM_SHAM_ (white) paradigm performed at the nondominant hand (pain-free, remote area). Data are presented as PPT values at each timepoint of the 2 paradigms (left panel) and as absolute parallel (PPT before − PPT during) and sequential (PPT before − PPT after) PPT changes in response to the 2 paradigms (ΔPPT; right panel). (B) PPT values before and during the cold water bath reflecting parallel CPM effects at the lower back (most painful area) and at the nondominant hand of CLBP patients (purple) and controls (turquoise). (C) Group comparison of relative parallel CPM effects (CPM ΔPPT), ie, ([PPT before − PPT during]/PPT before) × 100, at the lower back and at the nondominant hand. Negative absolute and relative ΔPPTs reflect inhibitory effects, and positive values reflect facilitatory effects. The dotted line depicts a null effect. The plots represent the raw data (coloured semitransparent dots), means (coloured dots), and 95% confidence intervals (black bars). **P < 0.01, ***P < 0.001. CLBP, nonspecific chronic low back pain; CPM, conditioned pain modulation; PPT, pressure pain threshold.

First testing for the presence of a “true” CPM effect beyond effects unrelated to the painfulness of the CS at the hand of the controls revealed a significant difference between the CPM and the CPM_SHAM_ effect on the PPTs (*V* = 278, *P* = 0.004, r = 0.59). Inhibition of PPTs was stronger during CPM compared with during CPM_SHAM_ indicating a “true” parallel CPM effect (Table 2, Fig. 4A). There was no difference in sequential CPM effects between the CPM and the CPM_SHAM_ paradigm (*V* = 212.5, *P* = 0.183, r = 0.26) (Table 2, Fig. 4A).

Further investigation of the “true” inhibitory parallel CPM effect showed that the inhibitory parallel CPM effect on PPTs was significant in both cohorts at the lower back (CLBP: *F* = 31.4, *P* < 0.001, controls: *F* = 30.6, *P* < 0.001) and hand (CLBP: *F* = 11.2, *P* = 0.002, controls: *F* = 13.8, *P* < 0.001) (Table 2, Fig. 4B). Parallel CPM effects were not different between CLBP patients and controls (Table 2, Fig. 4C).

The SEM for PPTs was 12.4%. Therefore, participants showing CPM effects beyond 24.8% were classified as CPM inhibitors (<−24.8%) or CPM facilitators (>24.8%). There was no difference in the proportions of CPM inhibitors (lower back: 47.5% of CLBP patients, 53.6% of controls; hand: 33.3% of CLBP patients, 55.6% of controls), CPM facilitators (lower back: 7.5% of CLBP patients, 3.6% of controls; hand: 2.6% of CLBP patients, 7.4% of controls), and CPM nonresponders (lower back: 45.0% of CLBP patients, 42.9% of controls; hand: 64.1% of CLBP patients, 37.0% of controls) between the cohorts (lower back: *P* = 0.794, hand: *P* = 0.142).

### 3.4. Associations of Glx/GABA with conditioned pain modulation effects and experimental pressure pain sensitivity

Associations of Glx/GABA with parallel CPM effects at the hand differed between the cohorts (*F* = 5.4, *P* = 0.046, *partial η*^*2*^ = 0.08). In controls, lower Glx/GABA was associated with larger CPM effects, but this association was not observed in CLBP patients (Supplementary Figure 4, http://links.lww.com/PAIN/C268). No cohort differences were observed for associations of Glx/GABA with parallel CPM effects at the lower back (Supplementary Table 2, Supplementary Figure 4, http://links.lww.com/PAIN/C268).

**Figure 5.**
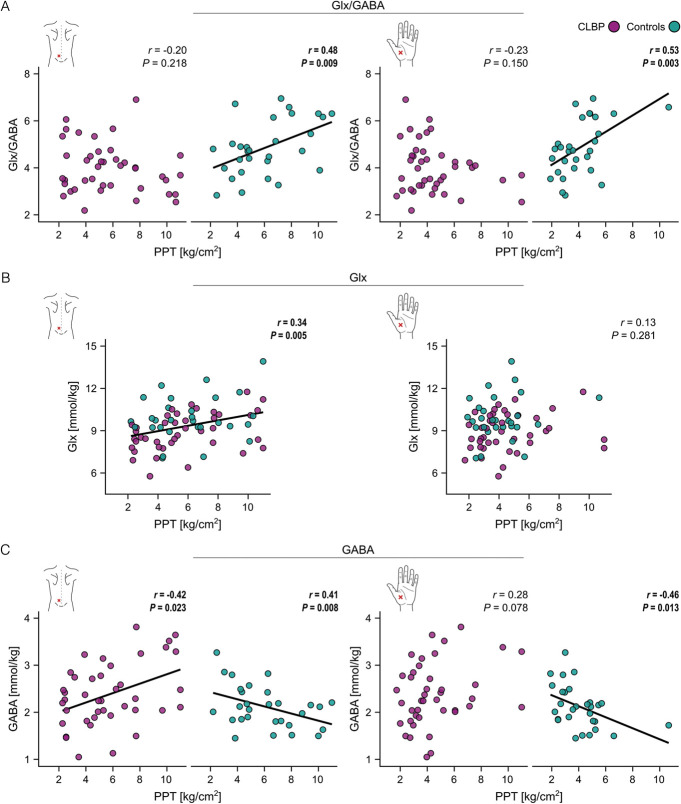
Associations of Glx/GABA, Glx, and GABA with experimental pressure pain sensitivity. (A-C) visualize the results of the linear models testing cohort differences in associations of Glx/GABA, Glx, and GABA with experimental pressure pain sensitivity, ie, PPTs. Pearson correlations reflect the models' effects of interest, ie, the “experimental pressure pain sensitivity × cohort” interaction effect (significant for Glx/GABA and GABA in the lower back and the nondominant hand) and the main effect of “experimental pressure pain sensitivity” (significant for Glx in the lower back). (A) shows Pearson correlations between Glx/GABA and PPTs at the lower back (most painful area) and at the nondominant hand (pain-free, remote area) for CLBP patients (purple) and controls (turquoise), (B) shows Pearson correlations between Glx and PPTs at both areas for the pooled sample (CLBP patients + controls), ie, main effect, and (C) shows Pearson correlations between GABA and PPTs at both areas for CLBP patients and controls. Solid lines represent significant Pearson correlations. CLBP, nonspecific chronic low back pain; PPT, pressure pain threshold.

Associations of Glx/GABA with PPTs differed between the cohorts at the lower back *and* the hand (lower back: *F* = 9.0, *P* = 0.004, *partial η*^*2*^ = 0.12; hand: *F* = 12.1, *P* = 0.002, *partial η*^*2*^ = 0.16). Again, controls showed associations (lower Glx/GABA correlated with lower PPTs) that were not present in CLBP patients (Fig. 5A). For Glx, associations of Glx with PPTs were not different between cohorts (lower back: *F* = 0.2, *P* = 0.633, *partial η*^*2*^ = 0; hand: *F* = 0.1, *P* = 0.775, *partial η*^*2*^ = 0). Instead, an association across cohorts was observed at the lower back, where participants with lower Glx exhibited lower PPTs (*F* = 8.2, *P* = 0.006, *partial η*^*2*^ = 0.12) (Fig. 5B). No association was observed at the hand (*F* = 2.1, *P* = 0.154, *partial η*^*2*^ = 0.02). For GABA, associations of GABA with PPTs differed between the cohorts at the lower back *and* the hand (lower back: *F* = 11.6, *P* = 0.001, *partial η*^*2*^ = 0.15; hand: *F* = 7.6, *P* = 0.007, *partial η*^*2*^ = 0.10). While controls displayed lower PPTs with increasing GABA, CLBP patients showed the opposite, ie, higher PPTs with increasing GABA (Fig. 5C).

Parallel CPM effects depended on experimental pressure pain sensitivity, ie, controls showed significantly smaller CPM effects with increasing PPTs, but exclusively at the hand (*rho* = 0.63, *P* < 0.001; lower back: *rho* = 0.22, *P* = 0.545).

### 3.5. Associations of Glx/GABA and conditioned pain modulation effects with clinical characteristics of chronic low back pain patients

Clinical characteristics are summarized in Table 1 and a heatmap of the patients' spatial pain extent patterns is displayed in Supplementary Figure 5, http://links.lww.com/PAIN/C268.

**Figure 6.**
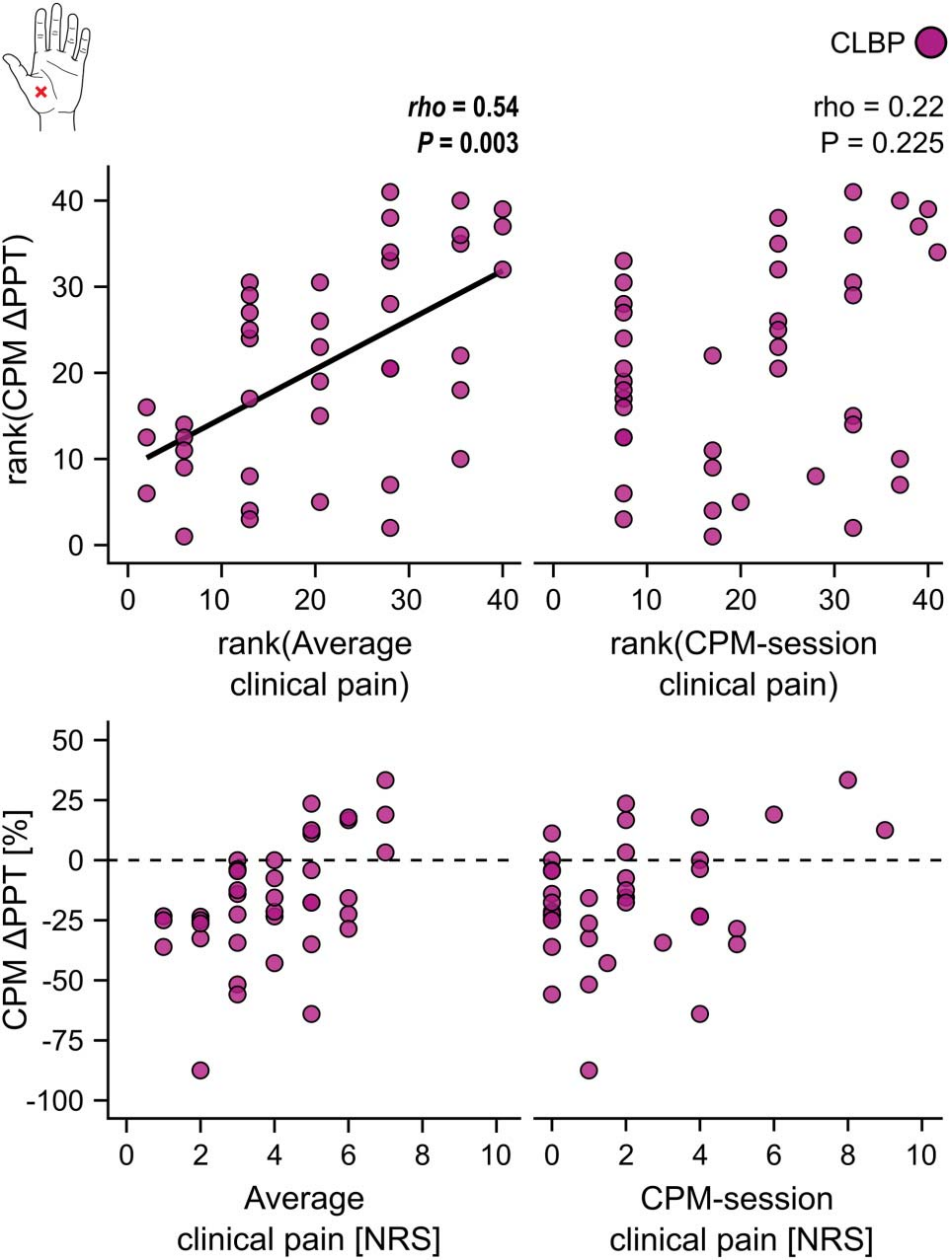
Associations of parallel CPM effects with clinical pain intensities. Spearman correlations between relative parallel CPM effects (CPM ΔPPT) and average clinical pain intensities over the past 4 weeks (average clinical pain), ie, “trait” pain, and within-CPM-session clinical pain (CPM-session clinical pain), ie, “state” pain. Upper panels show ranked data representative of Spearman correlations. To help with interpretability, the raw data are shown in the lower panels. Negative relative CPM ΔPPTs reflect inhibitory CPM effects, and positive relative CPM ΔPPTs reflect facilitatory CPM effects. The dotted line depicts a null CPM effect. CLBP, nonspecific chronic low back pain; CPM, conditioned pain modulation; NRS, numeric rating scale; PPT, pressure pain threshold.

Glx/GABA was not associated with any clinical characteristic, nor were Glx or GABA separately (Supplementary Table 6, http://links.lww.com/PAIN/C268). Smaller parallel CPM effects at the hand were observed for CLBP patients with higher average clinical pain intensities (*rho* = 0.54, *P* = 0.003), but not with higher within-CPM-session clinical pain (*rho* = 0.22, *P* = 0.225) (Fig. 6). No other associations between parallel CPM effects and clinical characteristics were observed (Supplementary Table 6, http://links.lww.com/PAIN/C268).

Given that parallel CPM effects at the hand depended on experimental pressure pain sensitivity (see the previous section), the correlation between parallel CPM effects at the hand and higher average clinical pain intensities could have been driven by the patients' experimental pressure pain sensitivity. However, average clinical pain intensities did not correlate with PPTs at the hand (*rho* = 0.20, *P* = 0.212).

In CLBP patients, a statistical trend was observed for associations between lower Glx/GABA and higher PCS (*rho* = −0.29, *P* = 0.066) and higher HADS anxiety scores (*rho* = −0.29, *P* = 0.061). Glx/GABA was not related to HADS depression scores (*rho* = −0.19, *P* = 0.244). For Glx and GABA separately, no associations were observed (all *rho*'s < 0.23, all *P*'s > 0.157).

### 3.6. Confounding factors

Neither menstrual cycle phase nor medication intake had an influence on Glx/GABA or parallel CPM effects in either area (Supplementary Results R2, http://links.lww.com/PAIN/C268).

## 4. Discussion

This study presents evidence for PAG dysfunction in patients with CLBP. First, CLBP patients showed a lower Glx/GABA ratio, ie, a lower excitatory/inhibitory balance, in the PAG compared with pain-free controls, driven by decreased Glx *and* increased GABA. Second, while controls showed a relationship between Glx/GABA and experimental pressure pain sensitivity, these associations were disrupted in the patients. In addition, CLBP patients with more severe clinical pain demonstrated reduced CPM capacities.

### 4.1. Lower Glx/GABA in the periaqueductal gray of chronic low back pain patients

Glx and/or GABA imbalances exist across chronic pain disorders.^60^ In the PAG, a higher inhibitory or lower excitatory tone^22,25^ might impede effective descending pain inhibition. This study aligns with preclinical evidence^25,29^ by showing lower Glx/GABA, driven by higher GABA and lower Glx in the PAG of CLBP patients. Of note, GABA and Glx are not limited to inhibitory and excitatory roles, respectively, and the observed results may reflect more complex metabolic alterations.

An earlier PAG ^1^H-MRS study reported higher Glx in patients with migraine.^76^ This discrepancy with the present results might be attributed to the migraine study's larger VOI that encompassed additional brainstem structures showing alterations in chronic migraine.^77^ Other PAG ^1^H-MRS studies did not describe Glx or GABA differences between patients and controls.^10,11,38,69^

The higher tCr and tCho in male CLBP patients and the increased mI in men compared to women align with the 3 metabolites being higher in men.^21^ In addition, these metabolites all increase with age^42^ and CLBP male patients were the oldest subgroup.

Within the VOI, CLBP patients showed lower WM and higher CSF tissue fractions with increasing age. This resembles age-related degenerative processes^31^ and might indicate accelerated brain aging in chronic pain.^17^ However, these factors did not drive the observed Glx or GABA differences because CSF tissue fractions were accounted for in the metabolite concentration calculations and neither Glx nor GABA were related to WM tissue fractions in the controls. Finally, Glx and GABA are predominantly present in GM.^45,50^

OVERPRESS and voxel-based flip angle calibration minimize 2 limitations of conventional PRESS sequences, namely chemical shift displacement artifacts and the susceptibility to B_1_ inhomogeneities. Although these steps improve spectral quality, they do not specifically address the limited reliability of GABA detection at 3T caused by spectral overlap. Nevertheless, the CRLBs of GABA were adequate, GABA concentrations in controls were similar to expected concentrations in human brain tissue,^24^ and no indication for an influence of spectral baseline or metabolite/lipid fits on GABA was observed. In addition, the achieved narrow spectral linewidths might have benefitted GABA quantification.^3,23,84^ Still, the observed GABA signals could include influences from molecules with similar resonance frequencies. MEscher-GArwood-PRESS^48^ sequences are more GABA-specific but unsuitable for the PAG because they require large VOI sizes (approximately 30 × 30 × 30 mm^361^) and are highly susceptible to frequency drifts.^26^ Achieving a high regional specificity required a long ^1^H-MRS acquisition time, increasing the risk of head motion. However, considering the participants' minimal head motion (∼10% of VOI size), the PAG very likely remained within the VOI throughout the acquisition.

### 4.2. Reduced conditioned pain modulation capacities in chronic low back pain patients with more severe clinical pain

Using a CPM_SHAM_ paradigm allowed for the detection of “true” CPM effects^33^ which were present for PPTs assessed during but not after the cold water bath. This can be explained by sequential CPM effects being smaller compared with parallel CPM effects due to decreasing CPM effects after cessation of the CS,^64^ as well as distraction effects contributing to parallel CPM effects.^80^ Nevertheless, the present results support the combination of PPT as TS and a cold water bath as CS as a robust CPM paradigm.^30,55^ Proportions of CPM inhibitors were similar to a previous study using the same CPM paradigm and TS changes beyond 2 SEM as classification criterion.^33^

Impaired CPM effects were not observed in the CLBP patients, opposing the notion of deficient descending pain modulation in chronic pain^1^ and some previous reports in CLBP.^16,52,62^ However, the recruited CLBP cohort reported relatively low average clinical pain intensities (median: NRS 4) and a meta-analysis found impaired CPM exclusively in low back pain patients with pain intensities above NRS 5.^46^ This is substantiated by the association observed here between higher average clinical pain intensities and smaller CPM effects at the hand. Importantly, CPM effects only correlated with “trait” pain but not “state” pain, indicating that CPM capacities relate to clinical pain and not to the sheer fact of being in pain during testing. Moreover, average clinical pain intensities were not associated with PPTs, suggesting that indeed CPM effects, and not “baseline” pain sensitivity, were related to clinical pain severity. The lacking association between CPM effects at the lower back and average clinical pain intensities was potentially due to the extra-segmental application of the CS, reported to induce larger CPM effects compared with segmentally applied CS.^34^ A similar pattern was observed here (CLBP/controls lower back: −25.8%/−33.0%; hand: −16.9%/−27.6%). These overall larger CPM effects might have masked pain-related CPM variations. Alternatively, CLBP patients may have a functional, protective descending pain inhibition of their lower back, but reduced capacities for the rest of their body.^75^

### 4.3. Glx/GABA correlates with experimental pressure pain sensitivity in controls but not in chronic low back pain patients

Unexpectedly, lower Glx/GABA was associated with larger CPM effects, ie, more pain inhibition, at the hand of the controls. However, lower Glx/GABA was also associated with lower PPTs, ie, higher pressure pain sensitivity, in both areas of the controls, supporting our hypothesis of lower Glx/GABA being related to reduced pain inhibition. Most likely, this contradictory finding resulted from interdependences between CPM effects and baseline PPTs. At the hand, controls with lower baseline PPTs showed larger *relative* CPM effects—a potential mathematical consequence, because the same absolute change will result in a larger relative change for lower baseline values. In addition, ceiling effects might occur in the case of high baseline PPTs because of the applied safety cut-off of 10 kg/cm^2^. At the lower back, PPTs were not correlated with CPM effects but Glx/GABA still correlated with PPTs. Thus, lower Glx/GABA in the PAG might rather be associated with lower PPTs than larger CPM effects. Given that the PAG is involved in (tonic) descending pain inhibition,^28^ shown to be particularly strong for deep tissue afferents,^82^ these results might reflect a weaker tonic PAG-driven descending inhibition of deep tissue afferents in pain-free individuals with lower Glx/GABA. Furthermore, as Glx/GABA was low in patients, low Glx/GABA could be an indicator of an individual's susceptibility to develop chronic pain.

**Figure 7.**
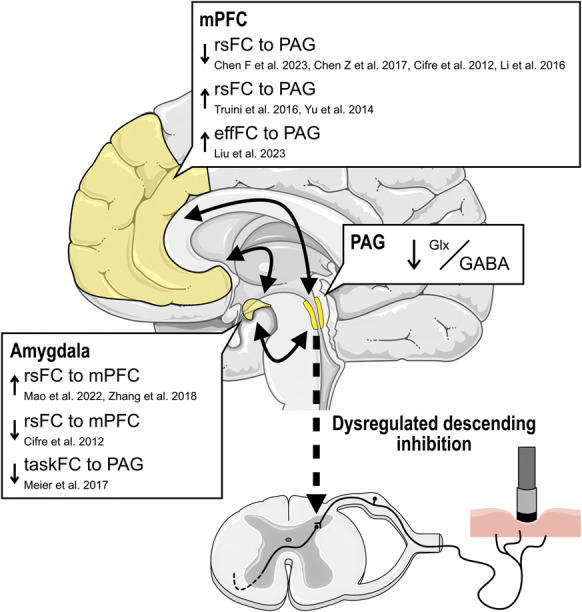
Clinical evidence for altered amygdala–medial prefrontal cortex (mPFC)–PAG connectivity patterns in chronic pain and potential consequences for PAG-driven descending pain inhibition. Summary of resting-state functional connectivity (rsFC) studies that found altered rsFC,^11,13,15,41,44,74,81,83^ altered effective rsFC (effFC),^43^ and altered task rsFC (taskFC; during observation of harmful activities)^47^ in the amygdala–mPFC–PAG circuit in chronic pain patients compared with pain-free controls. Altered supratentorial input to the PAG might be associated with dysregulated PAG metabolites and PAG-driven descending inhibition, such as the decreased Glx/GABA and the lacking association between Glx/GABA and experimental pressure pain sensitivity in CLBP patients observed in this study. Here, the anterior cingulate cortex was considered to be part of the mPFC.^57^ The schematic brain and spinal cord were adapted from Servier Medical Art (smart.servier.com). CLBP, nonspecific chronic low back pain; GABA, γ-aminobutyric acid; Glx, glutamate + glutamine; PAG, periaqueductal gray.

Chronic low back pain patients did not show associations between Glx/GABA and experimental pressure pain sensitivity and thus, their tonic PAG-driven descending inhibition of deep tissue afferents might be dysregulated. Associations between PAG metabolites and deficient descending pain inhibition have been suggested previously.^69^ PAG dysregulation might arise from altered supratentorial inputs such as the amygdala.^37^ Preclinical pain models show changes in amygdala–PAG pathways^40^ and increased amygdala activity resulting in decreased PAG activation through medial prefrontal cortex inhibition.^14^ Interestingly, multiple human functional connectivity studies have shown altered connectivity between these regions and the PAG in patients with chronic pain, including CLBP^11,13,15,41,43,44,47,74,81,83^ (Fig. 7). A possible association of Glx/GABA with amygdala-related factors is supported by the correlational trends observed here between Glx/GABA and PCS or HADS anxiety scores in CLBP patients.

The exploratory analyses of Glx and GABA separately showed that GABA was driving the dysregulated associations between Glx/GABA and PPTs in the patients. Interestingly, across cohorts, lower Glx was correlated with lower PPTs in the lower back. Taken together, the Glx and GABA results align with the concept of an increased GABAergic/decreased glutamatergic PAG tone relating to higher pain sensitivity^29^ and indicate a physiological Glx but a dysfunctional GABA function in the PAG of CLBP patients.

### 4.4. No further associations of Glx/GABA or conditioned pain modulation effects with clinical characteristics

Glx/GABA in CLBP was independent of clinical pain characteristics. As discussed above, CPM effects were smaller in CLBP patients with more severe clinical pain. Although previous reports documented reduced CPM capacities in CLBP patients with widespread pain,^22^ no such association was observed here. This might be due to the relatively low spatial pain extent in the recruited CLBP cohort.^19^

In summary, this study extends preclinical evidence by demonstrating a lower excitatory/inhibitory balance, ie, lower Glx/GABA, in the PAG of patients with CLBP. In addition, it shows a functional relevance of the PAG's excitatory/inhibitory balance, in that lower Glx/GABA was related to higher experimental pressure pain sensitivity in pain-free controls. Chronic low back pain patients lacked this association, indicating a dysregulated PAG function. Independent of PAG metabolites, CLBP patients with more severe clinical pain showed reduced CPM capacities. It remains to be investigated whether these alterations are CLBP-specific, related to etiologies including deep afferent-associated mechanisms, such as musculoskeletal conditions, or generally applicable to chronic pain states. Regardless, restoring the excitatory/inhibitory balance in the PAG might constitute a future treatment target for chronic pain and other conditions involving PAG dysfunction.

## Conflict of interest statement

The authors have no conflicts of interest to declare.

## Supplemental digital content

Supplemental digital content associated with this article can be found online at http://links.lww.com/PAIN/C268.

## Supplementary Material

SUPPLEMENTARY MATERIAL
