# Supplementary material for: The periaqueductal gray in chronic low back pain: dysregulated neurotransmitters and function
Source: Pain. 2025 May 15;166(7):1690–705. doi: 10.1097/j.pain.0000000000003617 (PMC12168810; doi:10.1097/j.pain.0000000000003617)
Supplement: SUPPLEMENTARY MATERIAL [file jop-166-1690-s001.pdf]

The periaqueductal gray in chronic low back pain: dysregulated neurotransmitters and function

Laura Sirucek,[1,2,3] Iara De Schoenmacker,[2,4,5] Lindsay Mary Gorrell,[1] Robin Lütolf,[4] Anke Langenfeld,[1] Mirjam Baechler,[1] Brigitte Wirth,[1] Michèle Hubli,[4] Niklaus Zölch,[6,7] and Petra Schweinhardt[1]

Author affiliations:

1 Department of Chiropractic Medicine, Balgrist University Hospital, University of Zurich, 8008 Zurich, Switzerland

2 Neuroscience Center Zurich, University of Zurich, 8057 Zurich, Switzerland

3 Center for Neuroplasticity and Pain (CNAP), Department of Health Science and Technology, Aalborg University, 9260 Aalborg, Denmark

4 Spinal Cord Injury Center, Balgrist University Hospital, University of Zurich, 8008 Zurich, Switzerland

5 Biomedical Data Science Lab, Institute of Translational Medicine, Swiss Federal Institute of Technology (ETH) Zurich, 8008 Zurich, Switzerland

6 Department of Forensic Medicine and Imaging, Institute of Forensic Medicine, University of Zurich, 8057 Zurich, Switzerland

7 Department of Psychiatry, Psychotherapy and Psychosomatics, Psychiatric Hospital, University of Zurich, 8032 Zurich, Switzerland

Supplementary Material

Supplementary Methods

M1. Sample size calculation

Sample size calculation based on expected effect sizes was not possible because to the best of our knowledge, at the time of protocol development, no study had examined Glx/GABA in the PAG. The sample size was thus chosen based on existing studies which showed differences in Glx or GABA between patients with different pain conditions and controls in other brain areas. Such MRS studies typically included around 19 participants per group.[1–20] Given the small size and the high level of physiological noise in the PAG, the aim was to recruit twice as many participants per group for the present study, i.e., 38 participants per group. In addition, due to

an expected higher variability in the CLBP patients compared to pain-free controls, a 20 % larger sample size for the CLBP cohort, i.e., 46 CLBP patients, was aimed for. For a two-tailed independent t-test with a 5 % probability to commit a type I error ($\alpha = 0.05$), this sample size results in a power of 0.95 for a large effect (0.8), a power of 0.62 for a medium effect (0.5) and a power of 0.15 for a small effect (0.2) (calculated using G*Power 3.1.9.7[21]).

M2. Confounding factors

Prior to the CPM and the ^{1}H-MRS session, information about the menstrual cycle phase was obtained if applicable, i.e., in premenopausal women not using menstruation-suppressing contraceptives.

In the electronic questionnaires, participants were asked to indicate any regular medication intake. Medications were classified according to the ATC/DDD classification by the World Health Organization (http://www.whocc.no/atc_ddd_index/). The following categories were considered to have potential influences on pain perception, referred to as pain-influencing medication: M01A (anti-inflammatory and anti-rheumatic drugs and non-steroids), N02 (analgesics), N03 (antiepileptics), N05 (psycholeptics), and N06 (psychoanaleptics). For ^{1}H-MRS, participants were also asked about ad hoc medication intake 24 h prior to MRS acquisition.

Proportions of the different menstrual cycle phases (menstruation, follicular, ovulation, or luteal) and of participants reporting regular or ad hoc pain-influencing medication intake were compared between the CLBP patients and controls using Fisher's exact tests.

For menstrual cycle phases, the proportions of participants in the different menstrual cycle phases (for 8 CLBPs and 6 controls: MRS session: *n* menstruation: 3 CLBP, 1 controls; *n* follicular: 4 CLBP, 3 controls; *n* luteal: 1 CLBP, 2 controls – CPM session: *n* menstruation: 2 CLBP, 2 controls; *n* follicular: 2 CLBP, 0 controls; *n* luteal: 3 CLBP, 3 controls; *n* ovulation: 1 CLBP, 1 controls) were not different between the 2 cohorts (Fisher's exact test: MRS session: $P = 0.790$, CPM session: $P = 0.773$) and data of the 2 cohorts were pooled to assess whether the menstrual cycle phase had an influence on Glx/GABA or the parallel CPM effects in both areas using Kruskal-Wallis tests (without multiple comparison correction to minimize the risk for false negatives).

For regular pain-influencing medication, the proportions of participants with/without regular pain-influencing medication intake (8/33 CLBP, 2/27 controls; 1 control used low dose aspirin as an anti-coagulant and one control used low dose quetiapine to improve sleep) were not different between the 2 cohorts (Fisher’s exact test: $P = 0.178$) and data of the 2 cohorts were

pooled to assess whether regular pain-influencing medication intake had an influence on Glx/GABA or the parallel CPM effects in both areas using Wilcoxon rank-sum tests (without multiple comparison correction to minimize the risk for false negatives). Additionally, separate Wilcoxon rank-sum tests were used to investigate whether such influences were present within the patient cohort. Controls with and without regular pain-influencing medication intake were not compared because of the small number of controls using regular pain-influencing medication.

For ad hoc pain-influencing medication, the proportions of participants with/without ad hoc pain-influencing medication intake (10/31 CLBP, 4/25 controls; 4 out of the 10 CLBP patients did not report regular pain-influencing medication intake, 1 control used low dose quetiapine to improve sleep, 3 controls used an NSAID, 1 due to a cold the morning before the measurement and 2 for unknown reasons the evening/night before the measurement) were not different between the 2 cohorts (Fisher's exact test: $P = 0.368$) and data of the 2 cohorts were pooled to assess whether ad hoc pain-influencing medication intake had an influence on Glx/GABA using a Wilcoxon rank-sum test. Additionally, a separate Wilcoxon rank-sum test was used to investigate whether an influence was present within the patient cohort. Controls with and without ad hoc pain-influencing medication intake were not compared because of the small number of controls using ad hoc pain-influencing medication.

M3. Model specifications

^{1}H-MRS outcomes:

Linear models (R package 'stats' with the function 'lm') were performed with the respective ^{1}H-MRS outcome as dependent variables and 'cohort' (levels: 'controls' and 'CLBP') as independent variable. For GABA, homogeneity of variance was not met and therefore, the 'cohort' effect was assessed using a Welch's t-test and age/sex influences were assessed in a separate linear model (for both cohorts combined because statistical inferences were not qualitatively different between the cohorts). For CSF tissue fractions, homogeneity of variance was not met and data was not normally distributed and therefore, the 'cohort' effect and sex influences were assessed using Wilcoxon rank-sum tests and age influences were tested using a Spearman correlation.

CPM effects:

Within-subject analyses: The presence of 'true' CPM effects beyond repeated-measures effects was tested on the data of the controls using two Wilcoxon signed rank tests (n-FDR: 2) because assumptions for a linear mixed model were not met. The dependent variable was the parallel or sequential CPM effect and the independent variable was the 'paradigm' (levels: 'CPM',

'CPMSHAM'). Age/sex effects were not assessed because age/sex-dependent differences between the CPM and CPMSHAM paradigm were not expected.

The linear mixed models (R package 'nlme', function 'lmer') assessing 'true' CPM effects in both cohorts in both areas were performed with PPT as dependent variable and 'timepoint' (levels: 'before', 'during', and 'after') as independent variable and the participants' identifier as random effect. Post-hoc tests were performed using planned comparisons ('before-during' and 'before-after'; R functions 'emmeans' followed by 'contrast') with Sidak's *P*-value adjustment. Age/sex influences were not assessed in these models because age/sex influences on CPM effects were tested in the models described below.

The 4 linear mixed models assessing 'true' CPM effects in both cohorts in both areas were performed with PPT as dependent variable and 'timepoint' (levels: 'before', 'during') as independent variable and the participants' identifier as random effect.

Between-subject analyses: The linear model assessing group differences in 'true' CPM effects in both areas were performed with the 'true' CPM effect as dependent variable and 'cohort' as independent variable. Only age/sex main effects were tested because age/sex interactions with cohort allocation had already been tested in the within-subject analyses.

Associations of Glx/GABA with CPM effects and experimental pressure pain sensitivity:

The linear models were performed with Glx/GABA as dependent variable and the 'true' CPM effect or the PPTs (proxy for experimental pressure pain sensitivity), 'cohort' and the interaction of interest ''true' CPM effect X cohort' or 'PPT X cohort' as independent variables.

M4. Statistical analysis of age/sex influences and assessment of influential cases

For all linear models, potential influences of age and sex on the dependent variables were examined by analyzing age and sex main effects and interaction effects with the model's independent variables. The models were always fitted with all interactions first, except for 'age X sex' interactions, which were not tested because the number of participants of different ages within a certain sex was low and this interaction was not of interest here. If the age/sex interactions were not significant, they were removed from the model to assess age/sex main effects. If the age/sex main effects were not significant, they were also removed from the model.

Influential cases are outliers in the model residuals which exert a large influence over the model.[22] Influential cases were identified based on standardized residuals for linear models[22]

and studentized residuals for linear mixed models,[23] and Cook's distance.[22] For standardized residuals, a cut-off of 1.96 was chosen.[22] For Cook's distance, a conservative cut-off of 4/(n-k-1)[24] (with n = number of observations and k = number of predictors in the model) was chosen to minimize false-positive or false-negative results driven by a small number of observations. For GABA, where the group comparison was made using a Welch's t-test, outliers > ±2.5 MAD were treated as influential cases.

M5. Spectral baseline and macromolecule/lipid fit comparison between cohorts

Spectral baseline and macromolecule/lipid fit were compared using one-dimensional Statistical Parametric Mapping (SPM: spm1d-package, www.spm1d.org).[25] This method is typically used for the analysis of voxel time-series obtained from functional MR imaging acquisitions.[26] However, it can also be utilized to analyze time-series of kinematic data.[25,27,28] For kinematic data, the advantage of this method is the ability to analyze entire kinematic curves rather than discrete (peak) values within these curves and to, for example, identify a time range in which kinematic curves differ between two cohorts.[27,29] In the context of the present study, this feature allowed to analyze chemical shift ranges in which spectral baseline and macromolecule/lipid fit differed between CLBP patients and controls. For that, data were first tested for normality using the D'Agostino K2 test (SPM function spm1d.stats.normality.k2.ttest). As data were non-normally distributed, a non-parametric, two-tailed, two-sample t-test with 10,000 permutations[30] was used to compare each datapoint of the spectral baseline and macromolecule/lipid fit across the chemical shift range between the cohorts, resulting in an output statistic SnPM{t} for each datapoint. Based on principles of random field theory validated for one-dimensional data,[31,32] a critical SnPM{t}-value threshold at which only 5% of smooth random curves would be expected to traverse, was considered statistically significant. Given that in time-series, as well as in MR spectra, neighboring points are dependent, the critical threshold is often exceeded by multiple adjacent points which are referred to as "suprathreshold clusters". Such suprathreshold clusters then characterize significant chemical range-specific differences between CLBP patients and controls.

To investigate whether identified differences in the spectral baselines (see Supplementary Results R1) could have caused the observed GABA differences between the cohorts, a total of 70 spectra (equal to the total number of participants) were simulated based on the FID-A generated basis set and using home-made MATLAB code. Identical metabolite concentrations (overall mean) were chosen for all simulated spectra, while the actual baseline, macromolecule/lipid contribution, and residual of each subject previously determined using

LC Model were included in the simulation. Additionally, to account for actually achieved spectral quality, the linewidths and SNR of the simulated spectra were adjusted to match the corresponding actual values for each subject.

M6. Standard error of measurement of pressure pain thresholds calculation

The standard error of measurement (SEM) for PPTs was calculated using data of all controls from the larger project which were measured at the hand as control area and randomized to perform the CPM_{SHAM} paradigm first (N = 30) with the formula:[33]

$$SEM\ [\%] = \frac{SD(PPT\ before\ CPM_{SHAM})\ x\ \sqrt{1 - ICC}}{mean(PPT\ before\ CPM_{SHAM})}\ x\ 100$$

The ICC (two-way, absolute agreement, single rater/measurement) was calculated between the PPT before and the PPT during CPM_{SHAM}. Participants were classified as CPM-inhibitors or CPM-facilitators if they presented with a PPT increase or PPT decrease, respectively, during CPM >2 SEM and otherwise as CPM-non-responders.[33]

Supplementary Results

R1. Spectral baseline and macromolecule/lipid fit comparison between cohorts

The spectral baseline was significantly lower in controls compared to CLBP patients in the chemical shift range between 1.59 ppm and 3.27 ppm (ClusterIntegral: 195.4, $P = 0.002$) (Supplementary Figure 1 A & B). The macromolecule/lipid fit did not differ between CLBP patients and controls (Supplementary Figure 1 C & D).

Given identical metabolite concentrations and considering the actual macromolecule/lipid contribution, residuals, and achieved spectral quality, the spectral baselines obtained in the participants of the present study did not result in GABA differences between cohorts based on the simulated spectra (simulated GABA/Cr of CLBP patients: 0.35 I.U., SD = 0.082; simulated GABA/Cr of controls: 0.35, SD = 0.069; unpaired t-test assuming equal variances: $t = -0.2$, $P = 0.833$).

R2. Confounding factors

Glx/GABA was not different between participants measured in different menstrual cycle phases ($\chi^2 = 1.5$, $P = 0.465$), nor between participants with or without regular pain-influencing medication intake ($W = 409$, $P = 0.069$), nor between patients with or without regular pain-influencing medication intake ($W = 178$, $P = 0.137$). Similarly, Glx/GABA was not different between participants with and without ad hoc pain-influencing medication intake (Wilcoxon rank-sum test: W = 477, p = 0.215), nor between patients with and without ad hoc pain-influencing medication (Wilcoxon rank-sum test: W = 193, p = 0.260). None of the participants used gabapentin or pregabalin (neither regularly nor ad hoc).

Also, the parallel CPM effects in both areas were not different between participants measured in different menstrual cycle phases (hand: $\chi^2 = 2.4$, $P = 0.497$; lower back: $\chi^2 = 0.9$, $P = 0.830$), nor between participants with or without regular pain-influencing medication intake (hand: $W = 244$, $P = 0.823$; lower back: $W = 276$, $P = 0.815$). Similarly, within the patient cohort, regular pain-influencing medication intake did not affect parallel CPM effects in either area (hand: $W = 85$, $P = 0.332$; lower back: $W = 128.5$, $P = 1$).

Supplementary Figures

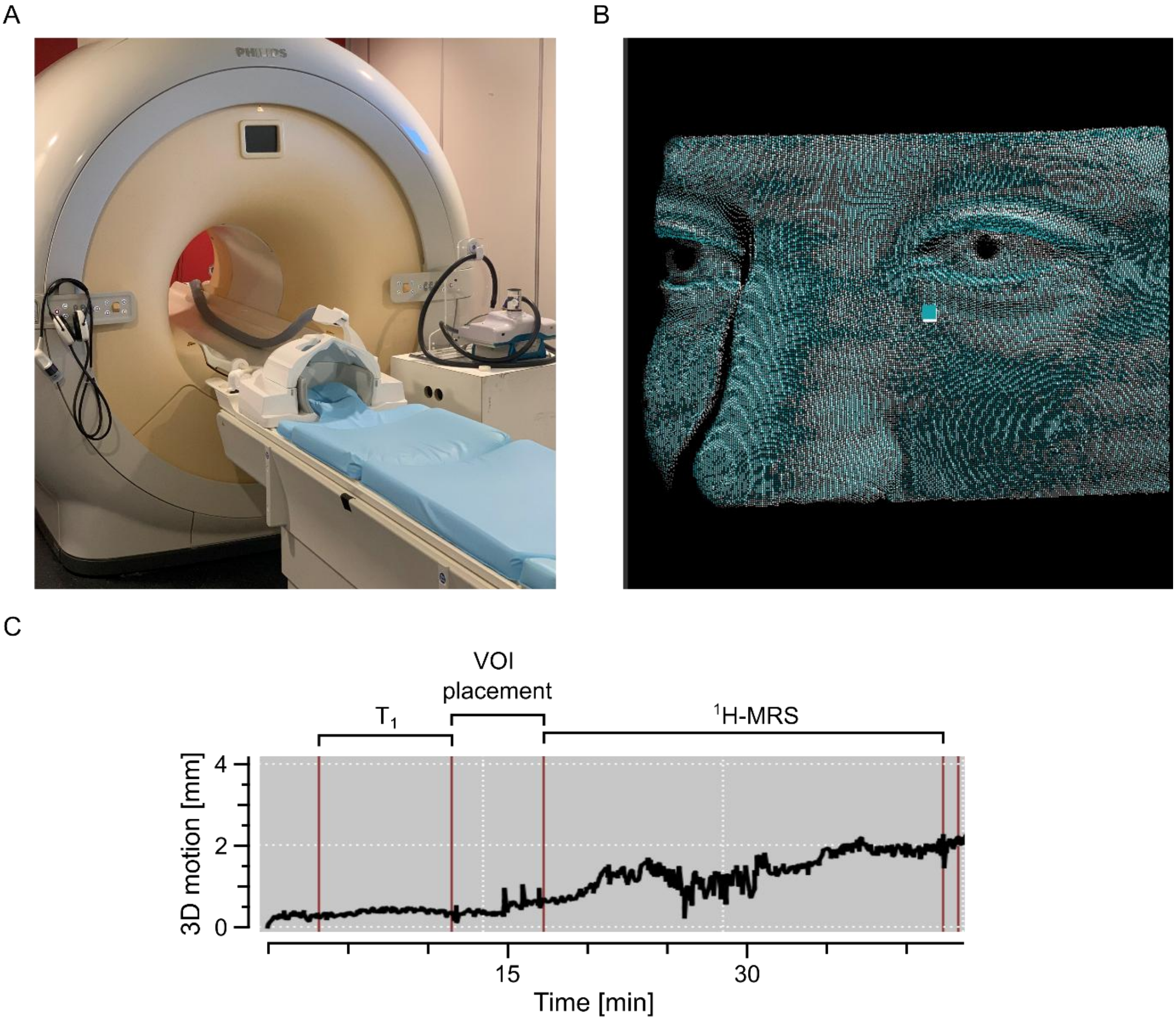

Supplementary Figure 1 Details on the markerless motion tracking system Tracoline TCL3 (TracInnovations, Ballerup, Denmark). The Tracoline is a 3D stereo vision system which uses structured invisible infrared light to construct 3D point clouds of the participant's face. (A) The set-up of the Tracoline system. (B) A 3D point cloud reconstruction example (for participant anonymity purposes of author LS's face). The TracSuite software estimates head motion by setting a reference point cloud to which subsequent point clouds are registered to. The calculated 3D motion represents the absolute motion of the point cloud's centroid point. (C) Representative absolute 3D motion recording. The beginning and the end of the ^{1}H-MRS acquisition in the periaqueductal gray was manually labeled during the scan (red lines) within the TracSuite software. T_1: anatomical T_1-weighted scan; VOI: volume of interest.

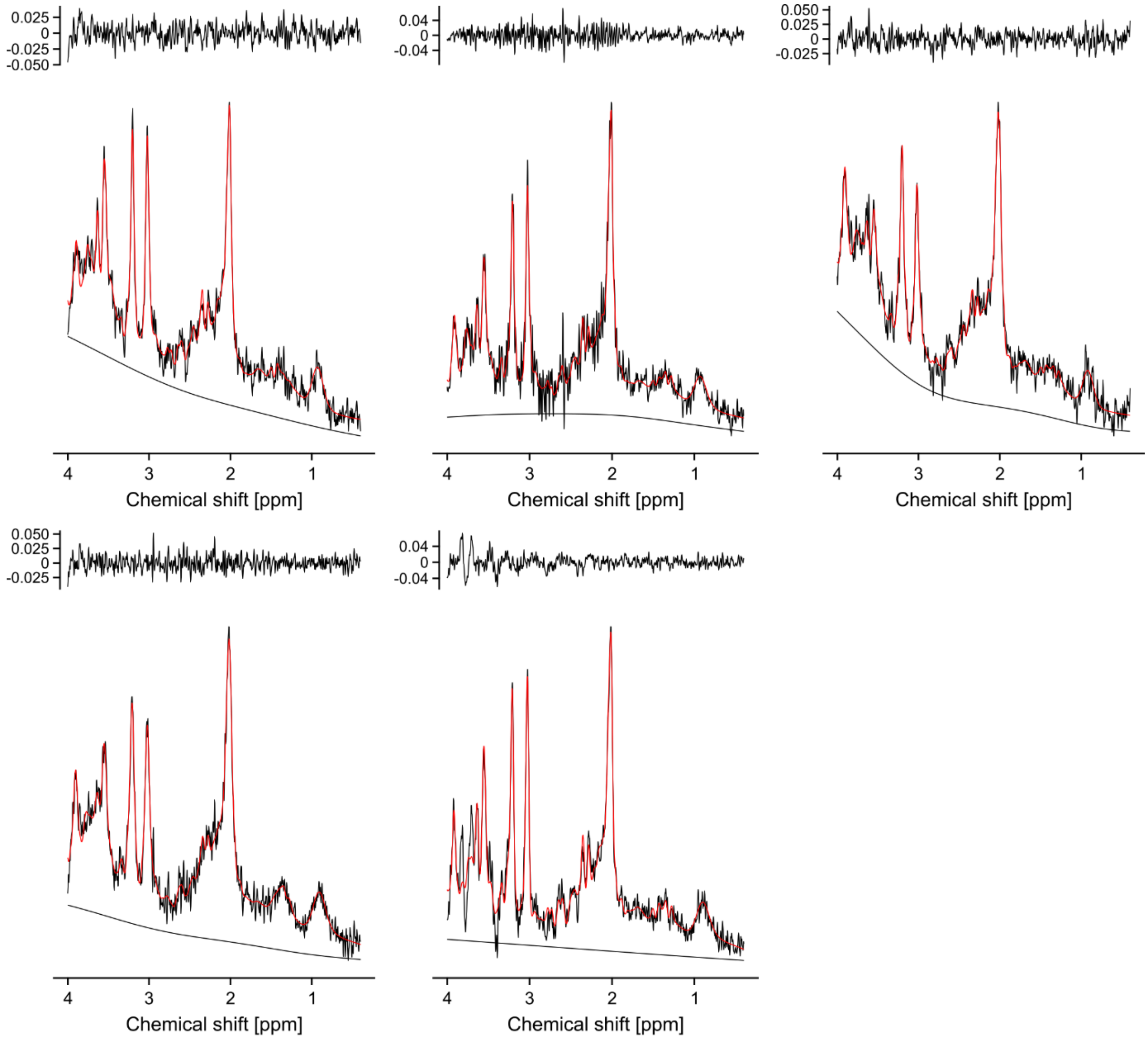

Supplementary Figure 2 Excluded ^{1}H-MR spectra due to the presence of artefacts. Spectra were excluded from 2 CLBP patients and 3 controls. Reasons for exclusion from top left to bottom right: distorted baseline; high frequency noise in frequencies of interest (3-2 ppm); distorted baseline; distorted baseline and high frequency noise in frequencies of interest; artefact in frequencies of interest (4-4.5 ppm). CLBP: non-specific chronic low back pain; ^{1}H-MR spectra: proton magnetic resonance spectra.

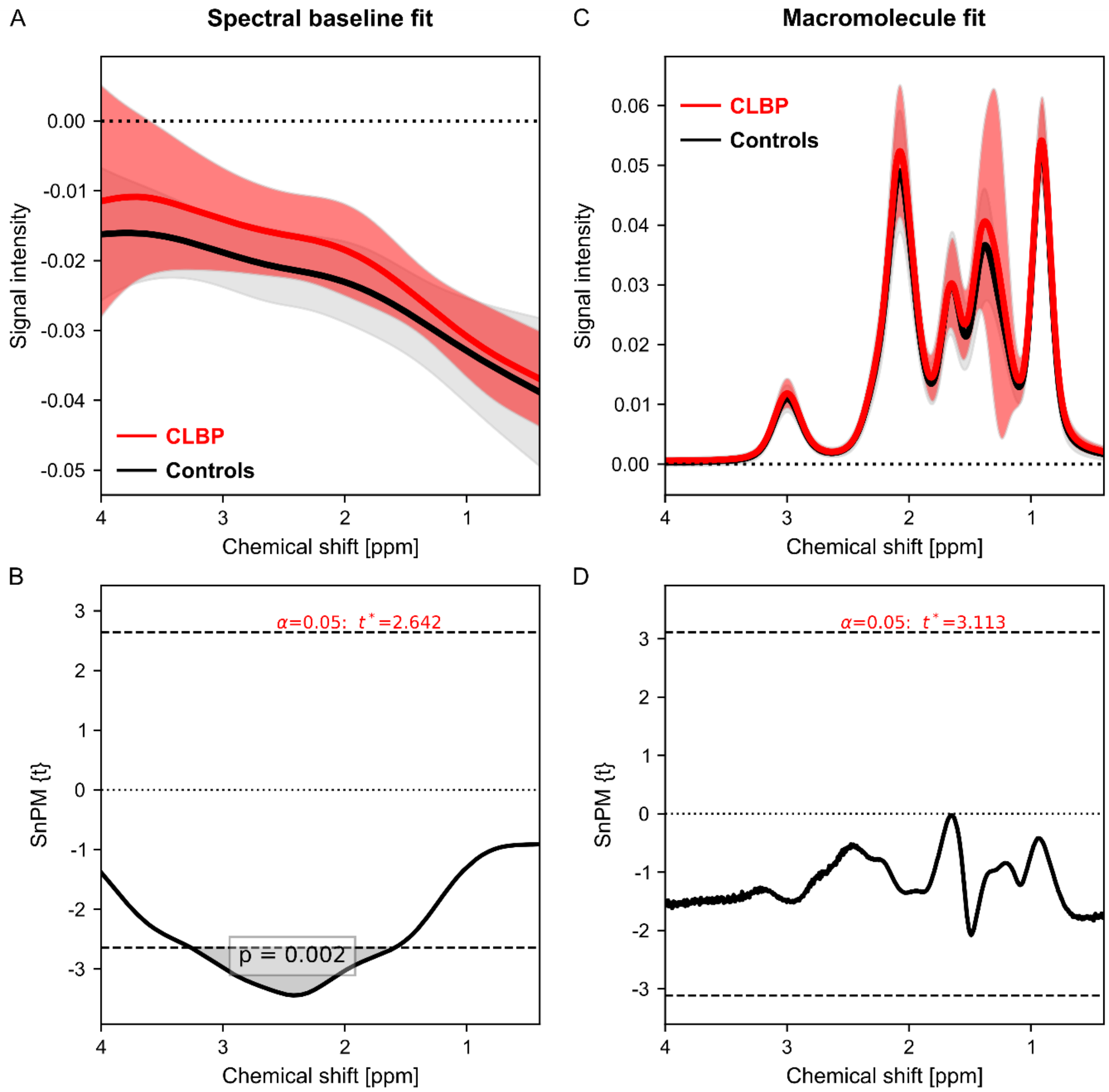

Supplementary Figure 3 Spectral baseline and macromolecule/lipid fit comparison between cohorts. (**A**) Mean spectral baseline fit and standard deviation (semi-transparent area) for CLBP patients (red) and controls (black). (**B**) SnPM{t}-values with suprathreshold clusters reflecting significant chemical range-specific differences in spectral baselines between CLBP patients and controls. (**C**) Mean macromolecule/lipid fit and standard deviation (semi-transparent area) for CLBP patients (red) and controls (black). (**D**) SnPM{t}-values with suprathreshold clusters reflecting significant chemical range-specific differences in macromolecule/lipid fit between CLBP patients and controls. CLBP: non-specific chronic low back pain.

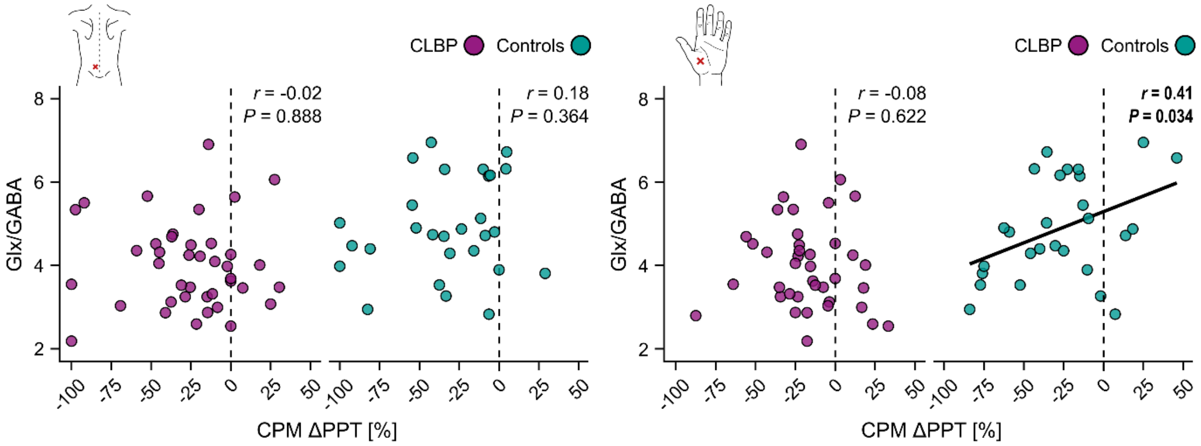

Supplementary Figure 4 Associations of Glx/GABA with 'true' CPM effects. The graphs visualize the results of the linear models testing cohort differences in associations of Glx/GABA with 'true' CPM effects. Pearson correlations reflect the models' interaction effect of interest, i.e., 'true' CPM effect X cohort' (significant for the non-dominant hand). The solid lines represent significant Pearson correlations between Glx/GABA and relative parallel CPM effects (CPM ΔPPT). Negative relative CPM ΔPPTs reflect inhibitory CPM effects, positive relative CPM ΔPPTs reflect facilitatory CPM effects. The dotted line depicts a null CPM effect. CLBP: non-specific chronic low back pain; CPM: conditioned pain modulation; PPT: pressure pain threshold.

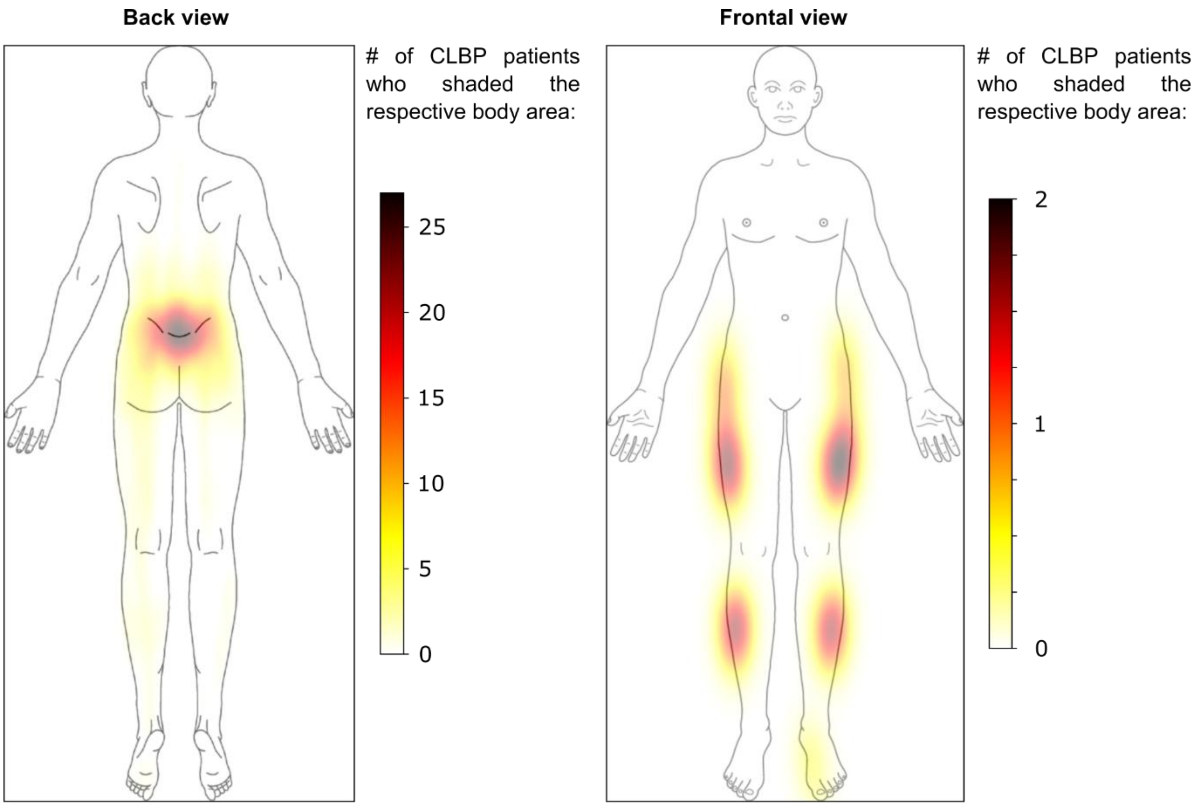

Supplementary Figure 5 Spatial pain extent heatmaps of CLBP patients (N = 41). Darker colors mark body areas which were more frequently reported to be painful. Out of 41 CLBP patients, 4 patients reported pain in the frontal half of the body. CLBP: non-specific chronic low back pain.

Supplementary Tables

Supplementary Table 1 Technical details of the ^{1}H-MRS acquisition using the experts' consensus checklist for a single voxel ^{1}H-MRS study.[34]

1. Hardware	
a. Field strength [T]	3 T
b. Manufacturer	Philips
c. Model (software version if available)	Achieva with dStream Upgrade. Software Release 5.6.1.
d. RF coils: nuclei (transmit/receive), number of channels, type, body part	32-channel dStream receive-only phased-array head coil
e. Additional hardware	N/A
2. Acquisition	
a. Pulse sequence	PRESS in combination with 6 saturation pulses (OVERPRESS)[35–37] and a voxel-based flip angle calibration performed prior to each measure-ment to achieve the desired flip angle and thus optimal SNR.[38,39]
b. Volume of Interest (VOI) locations	Periaqueductal gray
c. Nominal VOI size [cm3, mm3]	11x15x18 mm^3 (APxRLxFH). Accounting for the saturation pulses, the resulting effective VOI size was 8.8x10.2x12.2 mm^3 = 1.1 mL.
d. Repetition Time (TR), Echo Time (TE) [ms, s]	TR: 2500 ms, TE: 33 ms
e. Total number of Excitations or acquisitions per spectrum	512 acquisitions per spectrum divided into 8 blocks of 64 averages each. At the beginning of each block, 1 scan without water suppression was performed, resulting in a total of 8 acquisitions for the water reference (WR-shortTR).<br><br>Further, after completion of the 8 blocks, an additional water reference scan with TR 10000 ms was performed within the same VOI (WR-longTR). TEs were varied for the 6 acquired averages (+ 2 dummy scans), i.e., 33/66/107/165/261/600 ms, allowing to estimate the T_2 relaxation time of water within the VOI and therewith, obtain a subject-specific approximation of the fully-relaxed water signal within the VOI.[40] All settings were kept identical to the previous sequence except the center frequency of the applied pulses which was set to the resonance frequency of water instead of creatine.
f. Additional sequence parameters (spectral width in Hz, number of spectral points, frequency offsets)	Spectral width 2000 Hz, 2048 points.
g. Water Suppression Method	VAriable Power radiofrequency pulses with Optimized Relaxation delays (VAPOR)
h. Shimming Method, reference peak, and thresholds for "acceptance of shim" chosen	Second-order automatic pencil-beam shim shimming where pencil-beam excitations were performed trough a shim volume of 30x30x30 mm^3 aligned with the spectroscopy VOI (PB volume option).
3. Data analysis methods and outputs	
a. Analysis software	ReconFrame (GyroTools LLC, Zurich, Switzerland) to pre-process the spectra and LCModel version 6.3[41] for analysis. For the pre-processing, code from FID-A[42] was added to ReconFrame.
b. Processing steps deviating from quoted reference or product	Processing of .raw/.lab with ReconFrame including a) eddy current correction[43] and b) coil combination.<br><br>Then, for the processing with spectral registration: Frequency alignment using spectral registration in the time domain[44] (adopted from FID-A[42]). For the spectral registration in the time domain, data was filtered with a 2 Hz Gaussian filter. Only the first 500 ms were used for alignment and the single averages were aligned to the median of all averages.<br><br>Without spectral registration:<br>Minimal frequency alignment was achieved by the performed eddy current correction with the interleaved water unsuppressed scans (WR-shortTR scan).<br><br>Both approaches were followed by the following steps: c) residual water filtering, d) 1Hz Gaussian filtering, and e) measurement of FWHM of water peak (FWHM H2O) (Method 1 in FID-A[42]) using 8-fold zero- filling and taking the absolute value of the time domain water signal before fast Fourier transformation. Only the signal from the second unsuppressed water peak from WR-shortTR was used to determine FHWM H2O.
c. Output measure<br><br>(e.g., absolute concentration, institutional units, ratio)	Ratio to water signal from WR-shortTR or WR-longTR. Ratios were corrected for CSF fraction and multiplied with the inverse of the molecular weight of water. Relaxation attenuation of the

	metabolite signals was not corrected. With that, a rough estimate of moles of metabolite per mass of tissue water (excluding CSF) - molar concentration mol/kg, was achieved.<br><br>Based on the different WR scans, the fully relaxed water signal was estimated differently:<br><br>WR-shortTR:<br>The WR-shortTR water scan was provided as water reference to LCModel. Relaxation attenuation of the water signal was considered based on literature values. The following T_1 / T_2 values were used for the different tissue types (ms): GM: 1820 / 100; WM: 1080 / 70; CSF: 4160 / 500 and the following relative densities of Nuclear Magnetic Resonance-visible water: GM: 0.78, WM: 0.65, CSF: 0.97.<br><br>WR-longTR:<br>In this case the WR-longTR water scan was provided as water reference to LCModel. The fully-relaxed water signal was estimated based on the measured subject-specific TE series. The decay of the water was fitted with an exponential decay within MATLAB 2022 using "fitnlm" and used to estimate the water peak area at TE = 0 ms. The ratio of the water peak area at TE = 33 ms and TE = 0 ms was used to correct the conc. values resulting from LCModel.<br><br>General:<br>Relative tissue type volume fractions within the VOI were determined using the T_1-weighted planning images (three-dimensional magnetization-prepared rapid gradient-echo (MPRAGE) sequence.[45] 1mm^3 isotropic, TE = 3.7 ms, TR = 8.1 ms, TI = 1024 ms, shot interval = 3000 ms, field of view: 240x160x240 mm^3 (APxLRxFH), flip angle = 8°, scan time = 7 min 32 s) which were segmented using SPM12.[46]<br><br>For both approaches, based on WR-shortTR and based on WR-longTR, ratios to water signal were obtained from LCModel with WCONC = 55556 and ATTH2O = 1.
d. Quantification references and assumptions, fitting model assumptions	The unsuppressed water peak (WR-shortTR or WR-longTR) was used as reference.<br><br>A simulated basis set containing the following 20 metabolites was used to determine peak areas in the chemical shift range from 0.4 ppm and 4.0 ppm:<br>alanine, aspartate, glucose, creatine, phosphor-creatine, glutamine, glutamate, glycerol-phosphocholine, phosphocholine, lactate, myo-inositol, N-acetylaspartate, N-acetylaspartyl-glutamate, scyllo-inositol, glutathione, taurine, glycine, phosphoethanolamine, ascorbate, and γ-aminobutyric acid.<br><br>Basis set simulations were performed using FID-A[42] (https://github.com/CIC-methods/FID-A retrieved commit from 2022 05 01), an open-source software toolkit for the simulation and processing of MRS data. 2D simulations were carried out over the effective voxel size (assuming ideal saturation bands) with a spatial resolution of 40 × 40 points in the directions of the refocusing pulses and using the actual pulse shape of the refocusing pulses.<br><br>Simulated contribution of macromolecules and lipid signals were provided within LCModel.
3. Data quality	
a. Reported variables<br><br>(SNR, Linewidth (with reference peaks))	SNR and FWHM of the N-acetylaspartate peak obtained from the LCModel output. To assess the shim quality in the VOI, FWHM H_2O from the water reference scan (WR-shortTR) was determined.
b. Data exclusion criteria	Visual inspection of artifacts and spectra with FWHM H_2O values above 2.5 mean absolute deviance (MAD)[47] of the group median or SNR values below 2.5 MAD of the group median.
c. Quality measures of postprocessing Model fitting (e.g., CRLB, goodness of fit, SD of residual)	Absolute CRLBs[48] of selected metabolites, i.e., relative % CRLBs obtained from LCModel multiplied by the conc. values obtained from LCModel.
d. Sample Spectrum	Figure 1

AP: anterior- posterior; CRLB: Cramér-Rao lower bound; CSF: cerebrospinal fluid; FH: foot-head; FOV: field of view; FWHM: full width at half maximum; GM: gray matter; LR: left-right; NMR: nuclear magnetic resonance; PRESS: point-resolved spectroscopy; SNR: signal-to-noise ratio; TE: echo time; TI: inversion time; TR: repetition time; VOI: volume of interest; WM: white matter.

Supplementary Table 2 Linear model and Welch's test results with and without influential cases.

Dependent variable		*n*-IC	Independent variables											
**^{1}H-MRS outcomes**														
	**Influential cases**		*F*<br>**cohort**	*P*	*F*<br>**age**	*P*	*F*<br>**sex**	*P*	*F*<br>**cohort X age**	*P*	*F*<br>**cohort X sex**	*P*	*F*	*P*
Glx/GABA	with	1	10.7	**0.002**	2.6	0.114	0.6	0.442	0.0	0.965	2.6	0.115		
	without		13.6	**<0.001**										
Glx [mmol/kg]	with	4	8.0	**0.012**[a]	0.7	0.634[a]	2.9	0.155[a]	1.2	0.287	0.5	0.488		
	without		9.1	**0.007**[a]										
GABA[b] [mmol/kg]	with	1[†]	t = 3.8	0.055[a]	0.2	0.634[a]	2.1	0.155[a]						
	without		t = 4.5	**0.038**[a]										
tCr [mmol/kg]	with	4	not meaningful[c]		8.2	**0.006**	not meaningful[c]		0.4	0.525	6.5	**0.013**		
	without		not meaningful[c]		9.5	**0.003**	not meaningful[c]				17.5	**<0.001**		
tCho [mmol/kg]	with	3	not meaningful[c]		0.2	0.619	not meaningful[c]		0.4	0.513	6.5	**0.016**		
	without		not meaningful[c]				not meaningful[c]				6.0	**0.017**		
tmI [mmol/kg]	with	4	0.2	0.661	1.6	0.209	9.8	**0.003**	0.0	0.958	3.4	0.068		
	without		0.2	0.644			16.5	**<0.001**						
tNAA [mmol/kg]	with	4	0.2	0.654	2.7	0.107	0.4	0.529	3.3	0.075	2.2	0.141		
	without		0.0	0.949										
GM [% of VOI]	with	2[†]	4.0	**0.049**	0.0	0.984	0.3	0.611	0.2	0.674	0.0	0.903		
	without		4.0	0.050										
WM [% of VOI]	with	2	5.4	**0.024**	2.7	0.104	2.7	0.105	1.2	0.273	0.2	0.695		
	without		6.5	**0.013**										
CSF[d] [% of VOI]			*W* = 510	0.312	CLBP:<br>*rho* = 0.73<br>Con:<br>*rho* = 0.04	CLBP:<br>**<0.001**<br>Con:<br>0.831	CLBP:<br>*W* = 136<br>Con:<br>*W* = 83	CLBP:<br>0.056<br>Con:<br>0.405						
**CPM effects**														
			**timepoint**		**timepoint X age**		**timepoint X sex**							
PPT LB (CLBP) [kg/cm²]	with	2	31.4	**<0.001**[a]	3.5	0.142[a]	0.1	0.992[a]						
	without		30.4	**<0.001**[a]										
PPT Hand (CLBP) [kg/cm²]	with	2	11.2	**0.002**[a]	0.1	0.736[a]	0.0	0.992[a]						
	without		20.1	**<0.001**[a]										
PPT LB (controls) [kg/cm²]	with	1	30.6	**<0.001**[a]	4.8	0.076[a]	0.1	0.710[a]						
	without		48.5	**<0.001**[a]										
PPT Hand (controls) [kg/cm²]	with	3	13.8	**<0.001**[a]	2.6	0.119[a]	0.6	0.710[a]						
	without		23.3	**<0.001**[a]										

			cohort		age		sex							
CPM ΔPPT parallel LB [%]	with	4	0.8	0.388[a]	4.3	0.064[a]	2.6	0.217[a]						
	without		0.6	0.429[a]										
CPM ΔPPT parallel Hand [%]	with	3	2.3	0.274[a]	3.6	0.064[a]	0.2	0.670[a]						
	without		4.2	0.088[a]										
**Associations of Glx/GABA with CPM effects**														
**Lower back:**			**CPM ΔPPT parallel**		**CPM ΔPPT parallel X cohort**		**CPM ΔPPT parallel X age**		**CPM ΔPPT parallel X sex**		**CPM ΔPPT parallel X cohort X age**		**CPM ΔPPT parallel X cohort X sex**	
Glx/GABA	with	2	0.3	0.596[a]	0.7	0.409[a]	2.1	0.315[a]	2.1	0.197[a]	0.4	0.553[a]	0.5	0.942[a]
	without		0.1	0.707	2.2	0.147[a]								
**Hand:**			**CPM ΔPPT parallel**		**CPM ΔPPT parallel X cohort**		**CPM ΔPPT parallel X age**		**CPM ΔPPT parallel X sex**		**CPM ΔPPT parallel X cohort X age**		**CPM ΔPPT parallel X cohort X sex**	
Glx/GABA	with	1	1.9	0.354[a]	3.6	0.122[a]	0.3	0.598[a]	1.7	0.197[a]	0.5	0.553[a]	0.0	0.942[a]
	without		not meaningful[c]		5.4	**0.046[a]**								
**Associations of Glx/GABA, Glx, and GABA with experimental pressure pain sensitivity**														
**Lower back:**			**PPT**		**PPT X cohort**		**PPT X age**		**PPT X sex**		**PPT X cohort X age**		**PPT X cohort X sex**	
Glx/GABA	with	2	not meaningful[c]		9.0	**0.004[a]**	0.6	0.628[a]	0.1	0.786[a]	0.8	0.481[a]	1.6	0.408[a]
	without		not meaningful[c]		15.1	**<0.001[a]**								
Glx	with	2	8.2	**0.006[e]**	0.2	0.633[e]	1.4	0.241[e]	0.1	0.762[e]	0.0	0.838[e]	0.0	0.946[e]
	without		4.6	**0.036[e]**										
GABA	with	0	not meaningful[c]		11.6	**0.001[e]**	0.2	0.687[e]	0.0	0.940[e]	0.7	0.391[e]	1.6	0.215[e]
	without													
**Hand:**			**PPT**		**PPT X cohort**		**PPT X age**		**PPT X sex**		**PPT X cohort X age**		**PPT X cohort X sex**	
Glx/GABA	with	2	not meaningful[c]		12.1	**0.002[a]**	0.2	0.628[a]	0.1	0.786[a]	0.5	0.481[a]	0.5	0.468[a]
	without		not meaningful[c]		14.7	**<0.001[a]**								
Glx	with	2	2.1	0.154[e]	0.1	0.775[e]	0.8	0.367[e]	1.5	0.228[e]	1.9	0.171[e]	0.0	0.989[e]
	without		2.7	0.107[e]										
GABA	with	0	not meaningful[c]		7.6	**0.007[e]**	0.1	0.751[e]	0.0	0.872[e]	0.0	0.984[e]	0.7	0.420[e]
	without													

CLBP: non-specific chronic low back pain; Con: pain-free controls; CPM: conditioned pain modulation; CSF: cerebrospinal fluid; FDR: false discovery rate; GABA: γ-aminobutyric acid; Glx: glutamate + glutamine; GM: gray matter; ^{1}H-MRS: proton magnetic resonance spectroscopy; LB: lower back.; PPT: pressure pain threshold; tCr: creatine + phosphocreatine; tCho: glycerophosphocholine + phosphocholine; tmI: myo-inositol + glycine; tNAA; N-acetylaspartate + N-acetylaspartylglutamate; WM: white matter.

[a]FDR-corrected for *n* = 2 tests.

[b]Welch's test followed by linear model for age/sex influence assessment.

[c]Due to the presence of a significant interaction effect.

[d]Wilcoxon tests for cohort and sex differences and Spearman correlation for age influence assessment.

[e] No multiple comparison correction performed due to the exploratory nature of the analyses.*n*-IC[†]: statistical inference changed with removal of influential cases.

Supplementary Table 3 CRLBs of measured metabolites using ^{1}H-MRS.

	CLBP patients (*n* = 41)	Controls (*n* = 29)
**Absolute CRLBs[a]**		
Glx [I.U.]	82.9 (76.69 to 93.91)	85.2 (79.74 to 93.69)
GABA [I.U.]	58.5 (54.46 to 63.91)	59.3 (53.62 to 66.94)
tCr [I.U.]	21.1 (20.37 to 22.42)	22.3 (21.60 to 23.15)
tCho [I.U.]	7.8 (7.10 to 9.97)	7.9 (7.35 to 10.12)
tmI [I.U.]	39.4 (30.73 to 43.78)	37.6 (32.08 to 42.37)
tNAA [I.U.]	29.3 (28.07 to 31.06)	29.4 (28.00 to 30.56)
**Relative CRLBs**		
Glx [%]	6 (5 to 7)	5 (5 to 6)
GABA [%]	15 (14 to 19)	18 (15 to 20)
tCr [%]	2 (2 to 2)	2 (2 to 2)
tCho [%]	2 (2 to 3)	2 (2 to 3)
tmI [%]	3 (2 to 3)	3 (2 to 3)
tNAA [%]	2 (2 to 2)	2 (2 to 2)

Because not all outcome measures were normally distributed and to allow comparison between the CRLBs of different metabolites, all values are reported as median (interquartile range). CLBP: non-specific chronic low back pain; CPM: conditioned pain modulation; CRLB: Cramér-Rao lower bounds; GABA: γ-aminobutyric acid; Glx: glutamate + glutamine; ^{1}H-MRS: proton magnetic resonance spectroscopy; tCr: creatine + phosphocreatine; tCho: glycerophosphocholine + phosphocholine; tmI: myo-inositol + glycine; tNAA; N-acetylaspartate + N-acetylaspartylglutamate.

[a]Calculated by multiplying the relative CRLBs with the metabolite concentration as ratio to water from the LCModel output.[48]

Supplementary Table 4 Individual absolute and relative CRLBs of GABA.

Cohort	Absolute CRLB[a] [I.U.]	Relative CRLB [%]	Cohort	Absolute CRLB[a] [I.U.]	Relative CRLB [%]	Cohort	Absolute CRLB[a] [I.U.]	Relative CRLB [%]
Control	0.51	19	Control	0.62	15	CLBP	0.53	18
Control	0.51	16	Control	0.58	21	CLBP	0.69	15
Control	0.76	23	Control	0.64	14	CLBP	0.55	21
Control	0.59	18	Control	0.64	18	CLBP	0.52	16
Control	0.70	29	Control	0.55	20	CLBP	0.58	11
Control	0.53	17	CLBP	0.64	14	CLBP	0.48	22
Control	0.62	24	CLBP	0.49	22	CLBP	0.73	15
Control	0.54	21	CLBP	0.79	15	CLBP	0.48	13
Control	0.52	13	CLBP	0.57	14	CLBP	0.77	18
Control	0.54	14	CLBP	0.47	15	CLBP	0.56	11
Control	0.43	13	CLBP	0.55	20	CLBP	0.68	14
Control	0.50	19	CLBP	0.58	20	CLBP	0.50	28
Control	0.68	13	CLBP	0.60	21	CLBP	0.55	19
Control	0.58	15	CLBP	0.49	11	CLBP	0.54	20
Control	0.48	21	CLBP	0.45	16	CLBP	0.52	16
Control	0.59	17	CLBP	0.61	16	CLBP	0.67	13
Control	0.66	19	CLBP	0.55	15	CLBP	0.57	18
Control	0.47	20	CLBP	0.51	14	CLBP	0.48	16
Control	0.56	17	CLBP	0.57	24	CLBP	0.48	30
Control	0.49	11	CLBP	0.62	14	CLBP	0.57	14
Control	0.48	20	CLBP	0.52	13	CLBP	0.61	17
Control	0.71	30	CLBP	0.52	15	CLBP	0.67	13
Control	0.65	20	CLBP	0.52	14			
Control	0.71	16	CLBP	0.51	15			

CLBP: non-specific chronic low back pain; CRLB: Cramér-Rao lower bounds; GABA: γ-aminobutyric acid.
[a]Calculated by multiplying the relative CRLBs with the metabolite concentration as ratio to water from the LCModel output.[48]

Supplementary Table 5 ^{1}H-MRS outcomes using literature-based water signals for metabolite quantification.

	n-IC	CLBP patients (*n* = 41)	Controls (*n* = 29)	Test statistic	*P*	Effect size
**MRS outcomes**						
Glx/GABA	1	4.0 (1.05)	4.9 (1.17)	F = 10.7	**0.002**	η^2 = 0.14
Glx [mmol/kg]	4	8.3 (1.14)	9.3 (1.43)	F = 10.0	**0.005**[a]	η^2 = 0.13
GABA [mmol/kg]	2†	2.2 (0.62)	2.0 (0.43)	F = 4.3	**0.043**[a]	d = 0.44
tCr [mmol/kg]	2	6.1 (0.41)	6.2 (0.37)	F = 2.5	0.118	η^2 = 0.03
tCho [mmol/kg]	4	2.1 (0.18)	2.1 (0.16)	F = 0.0	0.962	η^2 = 0.00
tmI [mmol/kg]	3	8.3 (0.70)	8.4 (0.92)	F = 0.5	0.488	η^2 = 0.00
tNAA [mmol/kg]	3	8.5 (0.88)	8.4 (0.63)	F = 0.1	0.716	η^2 = 0.09

Values are presented as mean (SD). F-statistics refer to linear models or Welch's tests for GABA and NAA due to inhomogeneity of variance. η^2 values refer to *partial* η^2's. CLBP: non-specific chronic low back pain; FDR: false discovery rate; GABA: γ-aminobutyric acid; Glx: glutamate + glutamine; ^{1}H-MRS: proton magnetic resonance spectroscopy; tCr: creatine + phosphocreatine; tCho: glycerophosphocholine + phosphocholine; tmI: myo-inositol + glycine; tNAA; N-acetylaspartate + N-acetylaspartylglutamate.

[a]FDR-corrected for n = 2 tests.

n-IC†: statistical inference changed with removal of influential cases.

Supplementary Table 6 Associations of Glx/GABA and parallel CPM effects with clinical characteristics.

	Clinical characteristic	Test statistic	*P*	Missing values (*n*)
**Glx/GABA**	Average clinical pain intensity [NRS]	*rho* = -0.17	0.894[a]	0
	Pain duration [months]	*rho* = 0.05	0.965[a]	1
	Spatial pain extent [%]	*rho* = 0.12	0.894[a]	0
	Within-MRS-session clinical pain [NRS]	*rho* = 0.01	0.965[a]	0
Glx [mmol/kg]	Average clinical pain intensity [NRS]	*rho* = -0.03	0.863[c]	0
	Pain duration [months]	*rho* = 0.24	0.143[c]	1
	Spatial pain extent [%]	*rho* = 0.18	0.248[c]	0
	Within-MRS-session clinical pain [NRS]	*rho* = 0.16	0.332[c]	0
GABA [mmol/kg]	Average clinical pain intensity [NRS]	*rho* = 0.22	0.172[c]	0
	Pain duration [months]	*rho* = 0.13	0.437[c]	1
	Spatial pain extent [%]	*rho* = -0.06	0.692[c]	0
	Within-MRS-session clinical pain [NRS]	*rho* = 0.11	0.511[c]	0
**Parallel CPM effects**				
CPM △PPT parallel LB [%]	Average clinical pain intensity [NRS]	*rho* = 0.29	0.185[b]	1
	Pain duration [months]	*rho* = -0.32	0.185[b]	2
	Spatial pain extent [%]	*rho* = 0.17	0.327[b]	1
	Within-CPM-session clinical pain [NRS]	*rho* = 0.27	0.190[b]	1
CPM △PPT parallel Hand [%]	Average clinical pain intensity [NRS]	*rho* = 0.54	**0.003**[b]	2
	Pain duration [months]	*rho* = 0.10	0.545[b]	3
	Spatial pain extent [%]	*rho* = 0.18	0.327[b]	2
	Within-CPM-session clinical pain [NRS]	*rho* = 0.22	0.270[b]	2

Rho statistics refer to Spearman correlations. Missing values were omitted from the analyses. For pain duration, 1 participant did not indicate the month of pain onset. For CPM, values are missing due to time constraints during the water bath or examiner error. CPM: conditioned pain modulation; FDR: false discovery rate; GABA: γ-aminobutyric acid; Glx: glutamate + glutamine; LB: lower back; NRS: numeric rating scale; PPT: pressure pain threshold.
[a]FDR-corrected for *n* = 4 tests.
[b]FDR-corrected for *n* = 8 tests.
[c]No multiple comparison correction performed due to the exploratory nature of the analyses.

Supplementary Table 7 Number of missing values for ^{1}H-MRS outcomes, experimental pressure pain sensitivity and CPM effects.

	Missing values (n)	
	**CLBP patients (n = 41)**	**Controls (n = 29)**
SNR		
FWHM H_2O		
FWHM NAA		
Glx/GABA		
Glx		
GABA		
tCr	0	0
tCho		
tmI		
tNAA		
GM		
WM		
CSF		
**Experimental pressure pain sensitivity**		
PPT LB	0	0
PPT Hand	0	0
**CPM effects**		
	**CPM_{SHAM} Controls (n = 29)**	**CPM Controls (n = 29)**
PPT before Hand	0	1
PPT during Hand	0	1
PPT after Hand	0	0
△PPT parallel Hand	0	2
△PPT sequential Hand	0	1
	**CLBP patients (n = 41)**	**Controls (n = 29)**
Cold-water bath LB pain intensity	0	0
Cold-water bath Hand pain intensity	0	0
PPT before LB	0	0
PPT during LB	1	1
CPM △PPT parallel LB	1	1
PPT before Hand	0	1
PPT during Hand	2	1
CPM △PPT parallel Hand	2	2

For Wilcoxon rank-sum tests, t-tests or linear models, missing values were omitted from the analyses. For CPM within-subject analyses, linear mixed models accounted for missing values using maximum likelihood estimation. CPM values are missing due to time constraints during the water bath or examiner error. CLBP: non-specific chronic low back pain; CPM: conditioned pain modulation; CSF: cerebrospinal fluid; FWHM: full width at half maximum; GABA: γ-aminobutyric acid; Glx: glutamate + glutamine; GM: gray matter; ^{1}H-MRS: proton magnetic resonance spectroscopy; LB: lower back.; PPT: pressure pain threshold; SNR: signal-to-noise ratio; tCr: creatine + phosphocreatine; tCho: glycerophosphocholine + phosphocholine; tmI: myo-inositol + glycine; tNAA; N-acetylaspartate + N-acetylaspartylglutamate; WM: white matter.

References

1. Aguila, M. E. *et al.* Elevated levels of GABA+ in migraine detected using (1) H-MRS. *NMR Biomed* 28, 890–897 (2015).
2. As-Sanie, S. *et al.* Functional Connectivity is Associated With Altered Brain Chemistry in Women With Endometriosis-Associated Chronic Pelvic Pain. *The journal of pain: official journal of the American Pain Society* 17, 1–13 (2016).
3. Bridge, H. *et al.* Altered neurochemical coupling in the occipital cortex in migraine with visual aura. *Cephalalgia* 35, 1025–1030 (2015).
4. Fayed, N. *et al.* Brain dysfunction in fibromyalgia and somatization disorder using proton magnetic resonance spectroscopy: a controlled study. *Acta Psychiatr Scand* 126, 115–125 (2012).
5. Fayed, N., Andres, E., Viguera, L., Modrego, P. J. & Garcia-Campayo, J. Higher glutamate+glutamine and reduction of N-acetylaspartate in posterior cingulate according to age range in patients with cognitive impairment and/or pain. *Acad Radiol* 21, 1211–1217 (2014).
6. Fayed, N. *et al.* Localized 1H-NMR spectroscopy in patients with fibromyalgia: a controlled study of changes in cerebral glutamate/glutamine, inositol, choline, and N-acetylaspartate. *Arthritis Res Ther* 12, R134 (2010).
7. Feraco, P. *et al.* Metabolic abnormalities in pain-processing regions of patients with fibromyalgia: a 3T MR spectroscopy study. *AJNR Am J Neuroradiol* 32, 1585–1590 (2011).
8. Foerster, B. R. *et al.* Reduced insular gamma-aminobutyric acid in fibromyalgia. *Arthritis Rheum* 64, 579–583 (2012).
9. Gussew, A., Rzanny, R., Gullmar, D., Scholle, H. C. & Reichenbach, J. R. 1H-MR spectroscopic detection of metabolic changes in pain processing brain regions in the presence of non-specific chronic low back pain. *Neuroimage* 54, 1315–1323 (2011).
10. Gustin, S. M. *et al.* Thalamic activity and biochemical changes in individuals with neuropathic pain after spinal cord injury. *Pain* 155, 1027–1036 (2014).
11. Harper, D. E. *et al.* Relationships between brain metabolite levels, functional connectivity, and negative mood in urologic chronic pelvic pain syndrome patients compared to controls: A MAPP research network study. *Neuroimage Clin* 17, 570–578 (2018).
12. Harris, R. E. *et al.* Elevated insular glutamate in fibromyalgia is associated with experimental pain. *Arthritis Rheum* 60, 3146–3152 (2009).
13. Henderson, L. A. *et al.* Chronic pain: lost inhibition? *J Neurosci* 33, 7574–7582 (2013).

14. Ito, T. *et al.* Proton magnetic resonance spectroscopy assessment of metabolite status of the anterior cingulate cortex in chronic pain patients and healthy controls. *J Pain Res* 10, 287–293 (2017).
15. Niddam, D. M., Tsai, S. Y., Lu, C. L., Ko, C. W. & Hsieh, J. C. Reduced hippocampal glutamate-glutamine levels in irritable bowel syndrome: preliminary findings using magnetic resonance spectroscopy. *Am J Gastroenterol* 106, 1503–1511 (2011).
16. Siniatchkin, M. *et al.* Abnormal changes of synaptic excitability in migraine with aura. *Cereb Cortex* 22, 2207–2216 (2012).
17. Valdes, M. *et al.* Increased glutamate/glutamine compounds in the brains of patients with fibromyalgia: a magnetic resonance spectroscopy study. *Arthritis Rheum* 62, 1829–1836 (2010).
18. Widerstrom-Noga, E., Cruz-Almeida, Y., Felix, E. R. & Pattany, P. M. Somatosensory phenotype is associated with thalamic metabolites and pain intensity after spinal cord injury. *Pain* 156, 166–174 (2015).
19. Widerstrom-Noga, E. *et al.* Metabolite concentrations in the anterior cingulate cortex predict high neuropathic pain impact after spinal cord injury. *Pain* 154, 204–212 (2013).
20. Zielman, R. *et al.* Cortical glutamate in migraine. *Brain* 140, 1859–1871 (2017).
21. Faul, F., Erdfelder, E., Lang, A. G. & Buchner, A. G*Power 3: a flexible statistical power analysis program for the social, behavioral, and biomedical sciences. *Behav Res Methods* 39, 175–191 (2007).
22. Field, A., Miles, J. & Field, Z. *Discovering Statistics Using R*. (Sage publications, 2012).
23. Goode, K. redres: Redress Your Mixed Model Assumptions. https://goodekat.github.io/redres/index.html (2019).
24. Hair, J. F., Black, W. C., Babin, B. J. & Anderson, R. E. Advanced diagnostics for multiple regression. *Multivariate Data Analysis (7th Edition). Pearson Prentice Hall Publishing* (2009).
25. Pataky, T. C. Generalized n-dimensional biomechanical field analysis using statistical parametric mapping. *J Biomech* 43, 1976–1982 (2010).
26. Ashburner, J. SPM: a history. *Neuroimage* 62, 791–800 (2012).
27. Pataky, T. C., Robinson, M. A. & Vanrenterghem, J. Vector field statistical analysis of kinematic and force trajectories. *J Biomech* 46, 2394–2401 (2013).
28. Meier, M. L. *et al.* The impact of pain-related fear on neural pathways of pain modulation in chronic low back pain. *Pain Rep* 2, e601 (2017).
29. Papi, E., Bull, A. M. J. & McGregor, A. H. Alteration of movement patterns in low back pain assessed by Statistical Parametric Mapping. *J Biomech* 100, 109597 (2020).

30. Pataky, T. C., Vanrenterghem, J. & Robinson, M. A. Zero- vs. one-dimensional, parametric vs. non-parametric, and confidence interval vs. hypothesis testing procedures in one-dimensional biomechanical trajectory analysis. *J Biomech* 48, 1277–1285 (2015).
31. Pataky, T. C. RFT1D: Smooth one-dimensional random field upcrossing probabilities in Python. *J Stat Softw* 71, 1–22 (2016).
32. Pataky, T. C., Vanrenterghem, J. & Robinson, M. A. The probability of false positives in zero-dimensional analyses of one-dimensional kinematic, force and EMG trajectories. *J Biomech* 49, 1468–1476 (2016).
33. Kennedy, D. L., Kemp, H. I., Wu, C., Ridout, D. A. & Rice, A. S. C. Determining Real Change in Conditioned Pain Modulation: A Repeated Measures Study in Healthy Volunteers. *The journal of pain : official journal of the American Pain Society* 21, 708–721 (2020).
34. Lin, A. *et al.* Minimum Reporting Standards for in vivo Magnetic Resonance Spectroscopy (MRSinMRS): Experts' consensus recommendations. *NMR Biomed* 34, e4484 (2021).
35. Edden, R. A., Schar, M., Hillis, A. E. & Barker, P. B. Optimized detection of lactate at high fields using inner volume saturation. *Magn Reson Med* 56, 912–917 (2006).
36. Schulte, R. F., Henning, A., Tsao, J., Boesiger, P. & Pruessmann, K. P. Design of broadband RF pulses with polynomial-phase response. *J Magn Reson* 186, 167–175 (2007).
37. Wilson, M. *et al.* Methodological consensus on clinical proton MRS of the brain: Review and recommendations. *Magn Reson Med* 82, 527–550 (2019).
38. Deelchand, D. K., Kantarci, K. & Oz, G. Improved localization, spectral quality, and repeatability with advanced MRS methodology in the clinical setting. *Magn Reson Med* 79, 1241–1250 (2018).
39. Oz, G. *et al.* Advanced single voxel (1) H magnetic resonance spectroscopy techniques in humans: Experts' consensus recommendations. *NMR Biomed* e4236 (2020) doi:10.1002/nbm.4236.
40. Kreis, R., Ernst, T. & Ross, B. D. Absolute Quantitation of Water and Metabolites in the Human Brain. II. Metabolite Concentrations. *J Magn Reson B* 102, 9–19 (1993).
41. Provencher, S. W. Automatic quantitation of localized in vivo 1H spectra with LCModel. *NMR Biomed* 14, 260–264 (2001).
42. Simpson, R., Devenyi, G. A., Jezzard, P., Hennessy, T. J. & Near, J. Advanced processing and simulation of MRS data using the FID appliance (FID-A)-An open source, MATLAB-based toolkit. *Magn Reson Med* 77, 23–33 (2017).

43. Klose, U. In vivo proton spectroscopy in presence of eddy currents. *Magn Reson Med* 14, 26–30 (1990).
44. Near, J. *et al.* Frequency and phase drift correction of magnetic resonance spectroscopy data by spectral registration in the time domain. *Magn Reson Med* 73, 44–50 (2015).
45. Mugler 3rd, J. P. & Brookeman, J. R. Three-dimensional magnetization-prepared rapid gradient-echo imaging (3D MP RAGE). *Magn Reson Med* 15, 152–157 (1990).
46. Ashburner, J. *et al.* SPM12 Manual. *Functional Imaging Laboratory, Institute of Neurology* (2021).
47. Leys, C., Ley, C., Klein, O., Bernard, P. & Licata, L. Detecting outliers: Do not use standard deviation around the mean, use absolute deviation around the median. *J Exp Soc Psychol* 49, 764–766 (2013).
48. Kreis, R. The trouble with quality filtering based on relative Cramer-Rao lower bounds. *Magn Reson Med* 75, 15–18 (2016).
